# Diagnostic value of contrast-enhanced ultrasound for the depth of myometrial infiltration in early endometrial cancer: a meta-analysis

**DOI:** 10.3389/fonc.2025.1493246

**Published:** 2025-03-07

**Authors:** Siqi Li, Yingying Liang, Jiaxun Wang

**Affiliations:** Department of Ultrasound, The Affiliated Panyu Central Hospital, Guangzhou Medical University, Guangzhou, China

**Keywords:** endometrial cancer, contrast-enhanced ultrasound, meta-analysis, myometrial infiltration, diagnostic value

## Abstract

**Objectives:**

Globally, endometrial cancer (EC) is currently one of the most common gynecologic malignancies among females. Preoperative infiltration depth analysis is important for disease progression and prognostic impact. This study aimed to evaluate the diagnostic value of contrast-enhanced ultrasound (CEUS) in the infiltration depth analysis of EC.

**Method:**

Electronic databases PubMed, Embase, Cochrane Library, Web of Science, CNKI, Wanfang, and VIP were searched for more extensive literature on CEUS in the diagnosis of myometrial infiltration in EC patients up to March 29, 2024. Cochran Q and I² were used to assess the heterogeneity of eligible studies. Sensitivity (SEN), specificity (SPE), positive likelihood ratio (PLR), negative likelihood ratio (NLR), and diagnostic odds ratio (DOR) were analyzed for each clinical outcome using a bivariate random effects model. Summary receiver operating characteristic (SROC) curves were also generated.

**Results:**

In total, 23 papers with 1247 EC patients were included in the meta-analysis. The SEN, SPE, PLR, NLR, and DOR for the diagnosis of deep myometrial infiltration (DMI) of EC using CEUS were 0.84 [95% confidence interval (CI): 0.79, 0.89], 0.92 (95%CI: 0.90, 0.94), 11.05 (95%CI: 8.00, 15.25), 0.17 (95%CI: 0.12, 0.23), and 64.91 (95%CI: 37.11, 113.52), respectively. The area under the curve (AUC) was 0.95 (95%CI: 0.93, 0.97). For the diagnosis of superficial myometrial invasion (SMI) of EC by CEUS, the SEN, SPEN, PLR, NLR, DOR and AUC were 0.91 (95%CI: 0.85, 0.95), 0.80 (95%CI: 0.64, 0.90), 4.55 (95%CI: 2.34, 8.85), 0.11 (95%CI: 0.06, 0.21), 41.40 (95%CI: 12.14, 141.13), and 0.94 (95%CI: 0.91, 0.95), respectively.

**Conclusion:**

CEUS might be a reliable and practical technique for EC myometrial infiltration diagnosis. More clinical data and studies are still needed to confirm these results in the future.

## Introduction

Endometrial cancer (EC) has become the second most prevalent cancer in the female reproductive system worldwide (1). Over recent years, the incidence of EC has trended upward following the obesity epidemic, the aging of the population, and the increased use of hormone replacement therapies (2, 3). Generally, the prognosis for patients with EC is usually optimistic, with a 5-year survival rate of over 80% (4). However, deep myometrial infiltration (DMI, ≥50%) is a prognostic factor for lymph node metastasis and decreased survival in EC (5). Additionally, the depth of infiltration can dictate the extent of surgical resection and the selection of subsequent treatment options (6). For instance, lymph node dissection is of greater significance in patients with DMI than superficial myometrial invasion (SMI, <50%)/no myometrial infiltration (7). The myometrial infiltration is an essential predictor of tumor aggressiveness, prognosis, and treatment decision-making.

Based on conventional ultrasound, Contrast-Enhanced Ultrasound (CEUS) is a technique that enhances blood flow and tissue imaging through tiny bubbles of contrast (8). The endometrial microcirculation and perfusion characteristics can be visualized by CEUS, providing more detailed and objective imaging data for myometrial infiltration of EC (9). Histopathologic examination is considered the most reliable technique for assessing myometrial infiltration, but it is not suitable for early patient screening due to the characteristics of invasive and time-consuming (10). CEUS, as a new diagnostic tool for EC, has particular advantages: (1) It distinguishes between benign and malignant adnexal masses (11); (2) Compared with conventional imaging techniques such as Computed Tomography (CT) and Magnetic Resonance Imaging (MRI), it does not involve ionizing radiation and the contrast agent used is usually particles containing air bubbles, which is less risky, has fewer side effects, and is less expensive (12, 13). Recently, the utility of the CEUS in the diagnosis of EC has been reported (14–16). However, there is limited evidence from meta-studies on the diagnostic value of CEUS for EC. Geng et al. (17) and Tong et al. (18) studied the accuracy of CEUS in the diagnosis of EC and found that CEUS could be a valid method in the diagnosis and staging of EC. The diagnosis of the depth of infiltration of EC has an important impact on disease assessment, therapeutic decision-making, prognosis determination, and individualized treatment plan development. There is no diagnostic meta-analysis for EC infiltration depth by CEUS.

Herein, a meta-analysis was conducted to explore the value of CEUS in the early diagnosis of EC infiltrate depth. Accurate assessment of infiltrate depth can help improve patient survival and quality of life, and research can help determine disease assessment, treatment decisions, prognosis, and individualized treatment options.

## Methods

### Literature screening

An extensive search of electronic databases for records up to March 29, 2024, was performed on PubMed, Embase, Cochrane Library, Web of Science, China National Knowledge Infrastructure (CNKI), Wanfang database, and China Science and Technology Journal Database (VIP). The search formulas are attached in **Supplementary Table S1**.

### Standards of inclusion and exclusion

The inclusion and exclusion criteria were designed to ensure a high-quality and focused analysis of the diagnostic value of CEUS for assessing myometrial infiltration in early-stage EC (18). According to the PICO framework, the following detailed inclusion criteria were established (19): (P) Population: Studies must include patients diagnosed with early-stage endometrial cancer, specifically those undergoing assessment for myometrial infiltration. (I) Interventions: Studies that employed CEUS for evaluating myometrial infiltration depth were included. (C) Comparisons: For the assessment of deep myometrial infiltration (DMI, ≥50%), the control group included cases with either superficial infiltration (<50%) or no infiltration; for the assessment of superficial myometrial invasion (SMI, <50%), the control group consisted of patients with no infiltration. (O) Outcomes: Myometrial infiltration in EC was assessed using sensitivity (SEN), specificity (SPE), positive likelihood ratio (PLR), negative likelihood ratio (NLR), diagnostic odds ratio (DOR), and area under the curve (AUC). The inclusion criteria also required studies to meet specific quality thresholds. Only original research studies (prospective or retrospective) that directly evaluated the diagnostic accuracy of CEUS in assessing myometrial infiltration depth were included. The quality of eligible studies was evaluated using the QUADAS-2 tool (Quality Assessment of Diagnostic Accuracy Studies-2), and only studies with a low or moderate risk of bias were included. The analysis was limited to studies published in English or Chinese.

The criteria for exclusion in the study were as follows (20, 21): (1) Meta-analyses, reviews, animal trials, case reports, and conference abstracts. (2) Studies focusing on advanced-stage EC or other gynecological malignancies. (3) Studies with a high risk of bias, as assessed by the QUADAS-2 tool. (4) Studies with insufficient reporting of diagnostic performance metrics or missing data on CEUS outcomes. (5) Non-Chinese/non-English studies.

Studies with overlapping populations were handled carefully to avoid duplicate data. Overlap was identified by comparing study authors, recruitment institutions, study periods, sample sizes, and participant demographics. When overlapping populations were detected, the most recent study, the most complete dataset, or the study with the highest quality (as assessed by QUADAS-2) was included (22, 23). The rationale for excluding overlapping studies was documented in the **Supplementary Materials** to ensure transparency.

### Data extraction

Firstly, the data extraction form was designed according to the purpose of the study, which mainly included the following information: first author, year of publication, country, type of study, original study population, ultrasound modality, and contrast agent. Secondly, the retrieved articles were then subjected to duplicate literature removal in EndNote X9. Two independent reviewers assess the title and abstract of the duplicate-removed literature to ensure the studies is relevant to the study subject (24). Finally, the remained documents were downloaded in the full-text checking session for further review and content extraction. Any inconsistencies in the process of information extraction would be resolved by consulting with superiors and conducting discussions.

### Evaluation of the quality of the literature

To improve reliability in systematic evaluation and clinical practice, Quality Assessment of Diagnostic Accuracy Studies-2 (QUADAS-2) was applied to assess the quality of the included diagnostic accuracy studies (25). The assessment covered two main domains: the risk of bias and clinical applicability. Risk of bias was evaluated across four key areas: patient selection, index test, reference standard, flow and timing, while clinical applicability was assessed based in patient selection, index test, and reference standard. Each study was categorized as having a high, low, or unclear risk of bias. Studies were classified as “unclear risk” when critical methodological details were insufficiently reported. For instance, some studies did not specify whether patient selection was conducted consecutively or randomly, raising concerns about selection bias. Others lacked clear blinding procedures for CEUS interpretation or failed to define standardized diagnostic criteria, making it difficult to determine the reliability of the index test assessment (26). By identifying these limitations, this review enhances the transparency of the methodological quality of the included studies.

### Statistical analysis

Data were analyzed using Meta-disc (version 1.4), Stata (version 15.1), and Revman (version 5.4). Detecting and adjusting for threshold effects was an important step to ensure the reliability and clinical applicability of results. A strong positive Spearman correlation between the logarithm of SEN and the logarithm of (1-SPE) indicated a threshold effect (27). Cochran Q and I (2) statistics were used to assess heterogeneity across studies.

To address potential sources of heterogeneity, a bivariate random-effects model was employed for the analysis of SEN, SPE, PLR, NLR, DOR, AUC (28). Subgroup analyses were conducted to explore variations in diagnostic performance based on key study characteristics, such as imaging protocols, patient demographics, and study design. Additionally, sensitivity analyses were performed to evaluate the robustness of the results by systematically excluding studies with a high risk of bias or small sample sizes (29).

Publication bias was evaluated by Deeks’ funnel plots for outcomes with more than 9 articles (30), and the difference was considered statistically significant at *P <*0.05. These methodological approaches enhance the transparency and reliability of the findings while ensuring a more comprehensive evaluation of CEUS’s diagnostic value for myometrial infiltration in early EC.

## Results

### Characteristics of the included studies

**Figure 1:**
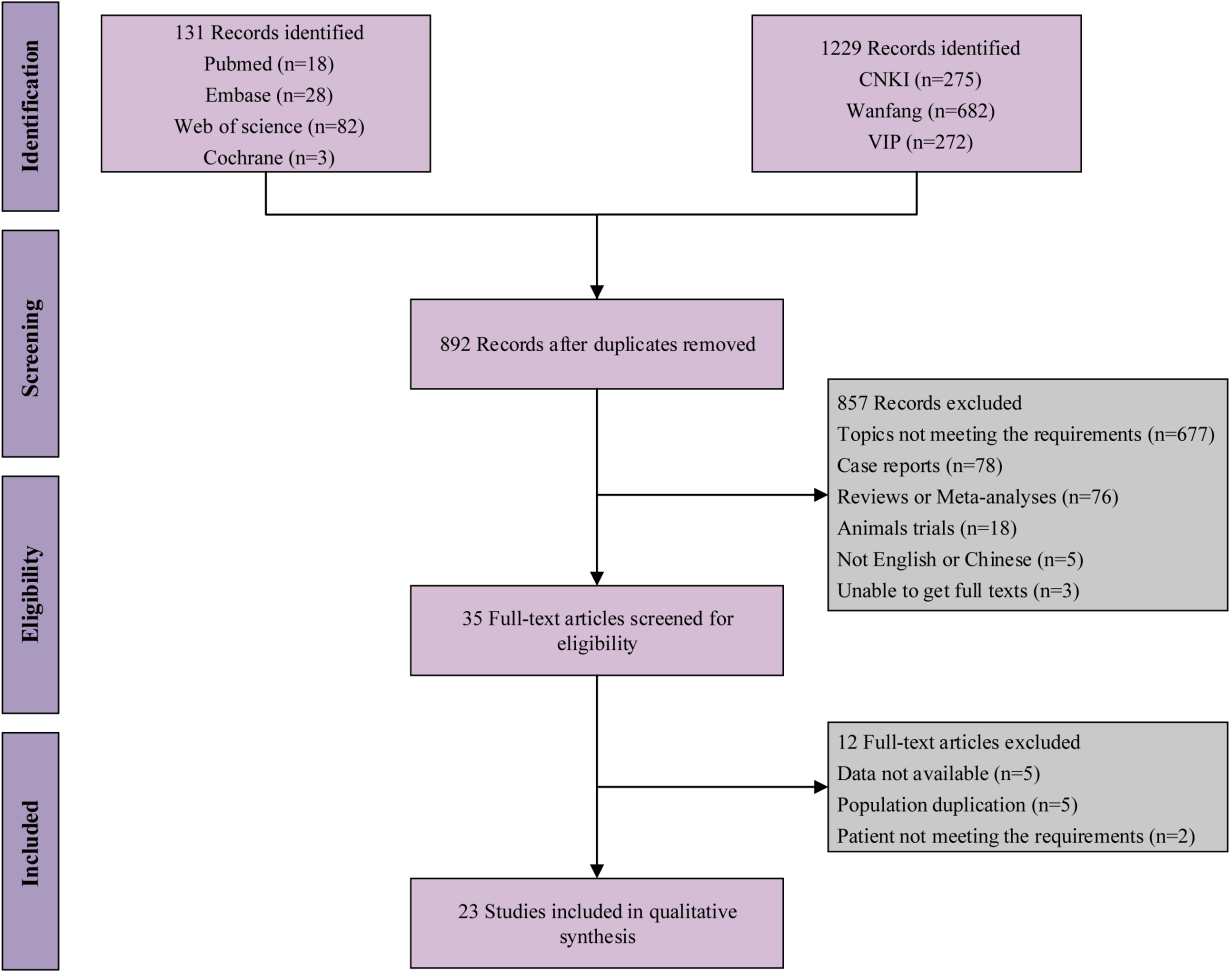
Flow chart of eligible studies.

After searching the databases according to the search strategy, 1,360 documents were identified. After screening titles and abstracts based on predetermined inclusion-exclusion criteria, 857 studies were excluded. After examining the 35 publications retained, 23 studies (14–16, 31–50) were included in the quantitative synthesis (**Figure 1**).

**Table 1** demonstrates the basic characteristics of the included studies. All articles were published between 2008 and 2023, with 1,247 EC patients recruited in the studies. The age range of the study population was 42.00 - 59.70 years. Furthermore, only one study employed greyscale, whereas the others used color Doppler to measure blood flow in the lesions.

**Table 1:** The characteristics of the included studies.

Author	Year	Study period	Design	Sample size (N)	Age (year)	Menstrual status (Pre-M/Post-M)	Approaching	Modality	Contrast agent	Volume of Contrast agent (mL)
Ben (13)	2022	2019.01-2021.12	–	120	51.47 ± 9.72	55/65	Transvaginal	Color Doppler	–	2.4
Chen (14)	2022	2020.02-2021.08	–	27	50.63 ± 2.75	4/8/15	Transvaginal	Color Doppler	SonoVue	2.5
Ding (22)	2013	2009.12-2012.08	Retrospective	40	55.00 (37.00-73.00)	27/13	Transabdominal	Color Doppler	SonoVue	2.4
Du (23)	2018	2012.05-2015.04	–	50	51.40 ± 7.50	–	Transvaginal	Color Doppler	SonoVue	2.4
Huang (24)	2016	2012.01-2015.02	–	80	49.80 ± 6.50	29/51	Transvaginal	Color Doppler	SonoVue	–
Huang (25)	2015	2012.05-2014.05	–	43	47.50 ± 14.80	27/16	Transvaginal	Color Doppler	SonoVue	–
Li (26)	2020	2019.03-2020.02	Retrospective	106	52.17 ± 6.83	26/80	Transvaginal	Color Doppler	SonoVue	–
Lin (27)	2022	2017.01-2020.12	–	48	51.40 ± 10.82	21/27	Transvaginal	Color Doppler	SonoVue	2.4
Lu (28)	2019	2017.01-2018.12	–	80	66.92 ± 2.51	12/68	Transvaginal	–	SonoVue	1.5
Mao (29)	2019	2015.11-2017.07	–	62	55.00 (25.00-75.00)	–	Transvaginal	Color Doppler	SonoVue	2.0
Ni (30)	2023	2015.12-2019.12	Retrospective	63	52.67 ± 16.28	–	–	Color Doppler	–	2.4
Pei (31)	2011	2009.01-2010.07	–	48	52.60 ± 1.40	–	Transabdominal	Color Doppler	SonoVue	1.0
Shabiti (32)	2015	2012.01-2014.01	–	60	55.40 ± 10.60	12/48	–	Color Doppler	SonoVue	2.4
Song (33)	2009	2006.06-2007.12	Prospective	35	–	–	Transvaginal	Grey-scale	SonoVue	2.4
Su (34)	2018	2011-2016	–	39	58.43 ± 7.12	13/26	Transvaginal	Color Doppler	SonoVue	2.4
Sun (21)	2008	2005.06-2007.12	Retrospective	30	55.40 (34.00-77.00)	6/24	–	Color Doppler	–	2.4
Tian (35)	2021	2019.12-2020.12	Retrospective	14	55.43 ± 6.12	4/10	Transvaginal	–	SonoVue	1.8-2
Wang (36)	2013	2011.09-2013.02	–	31	56.00	14/17	Transvaginal	Color Doppler	–	
Xie (37)	2012	2008.01-2010.10	–	37	56.00 (34.00-77.00)	9/28	–	Color Doppler	SonoVue	2.4
Xu (38)	2013	2009.10-2010.12	Retrospective	47	42.00 ± 11.00	11/36	Transvaginal	Color Doppler	SonoVue	2.4
Ye (15)	2023	2018.01-2021.01	Retrospective	65	59.70 ± 4.15	–	Transvaginal	Color Doppler	SonoVue	3.5
Zhang (39)	2018	2015.08-2017.04	Retrospective	82	54.58 ± 4.93	–	–	Color Doppler	SonoVue	2.4
Zou (40)	2013	2010.07-2013.03	–	40	52.00 (33.00-69.00)	15/25	Transvaginal	Color Doppler	SonoVue	2.4

Data are presented as mean ± standard deviation or median (range) for the age variable. Menstrual status is presented as premenopausal/postmenopausal or premenopausal/Perimenopausal/postmenopausal. Pre-M, Premenopausal; Post-M, Postmenopausal.

### Quality evaluation and research bias

As depicted in **Supplementary Figure S1A**, the quality assessment criteria for the studies included are categorized as either low-risk or unclear-risk. Within the risk of bias assessment, the Reference Standard contributed the most to the unclear-risk category, with Flow and Timing following behind. As for the Applicability Concerns of the included studies, all studies were shown to be low-risk. Studies including Chen et al. (15), Ding et al. (32), Lin et al. (37), Pei et al. (41), and Zou et al. (50) were considered relatively low-risk (**Supplementary Figure S1B**).

### Diagnostic value of CEUS in EC with DMI

**Figure 2:**
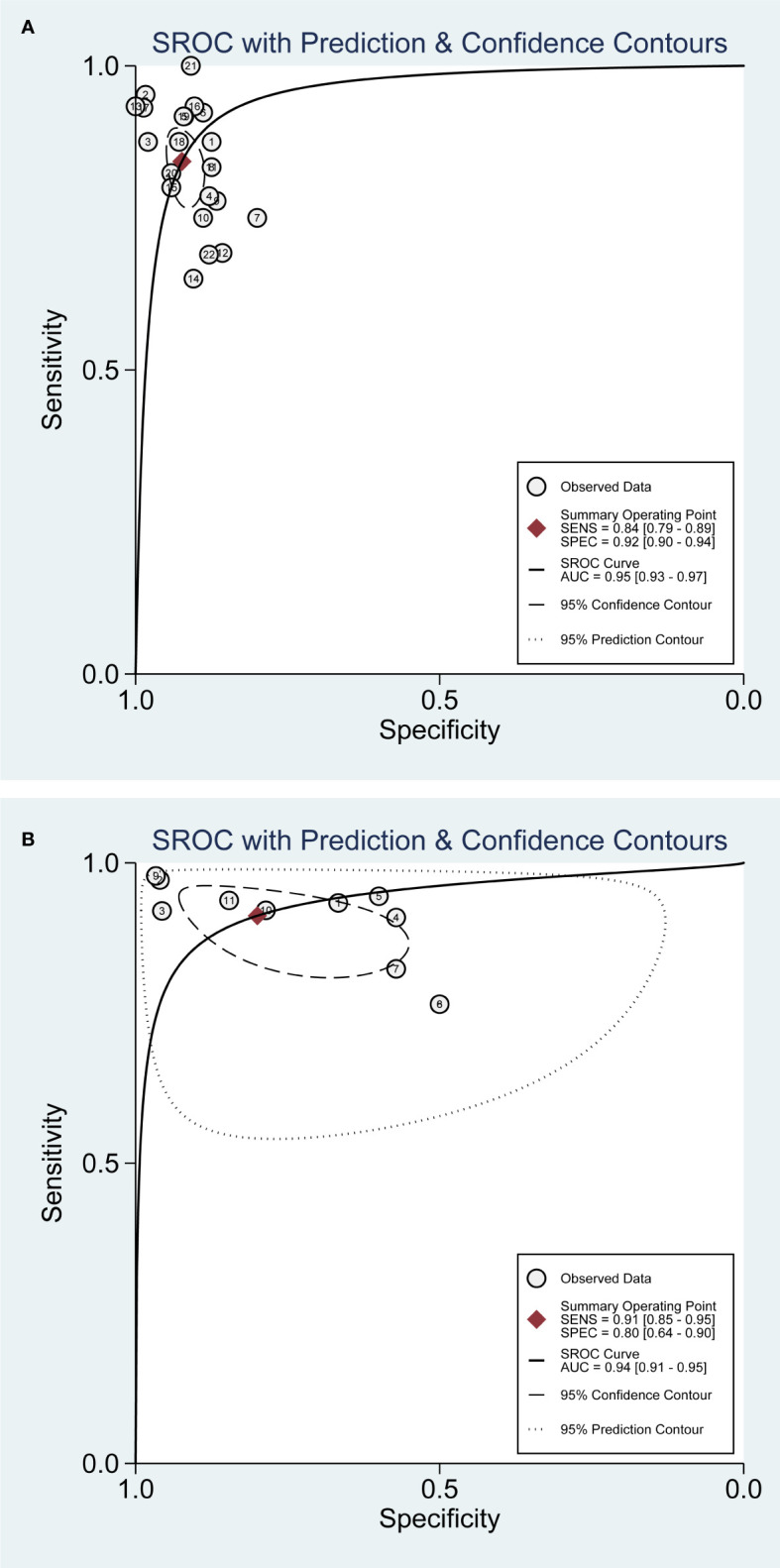
SROC curves for CEUS diagnosis of EC myometrial infiltration. **(A)** DMI; **(B)** SMI.

**Figure 3:**
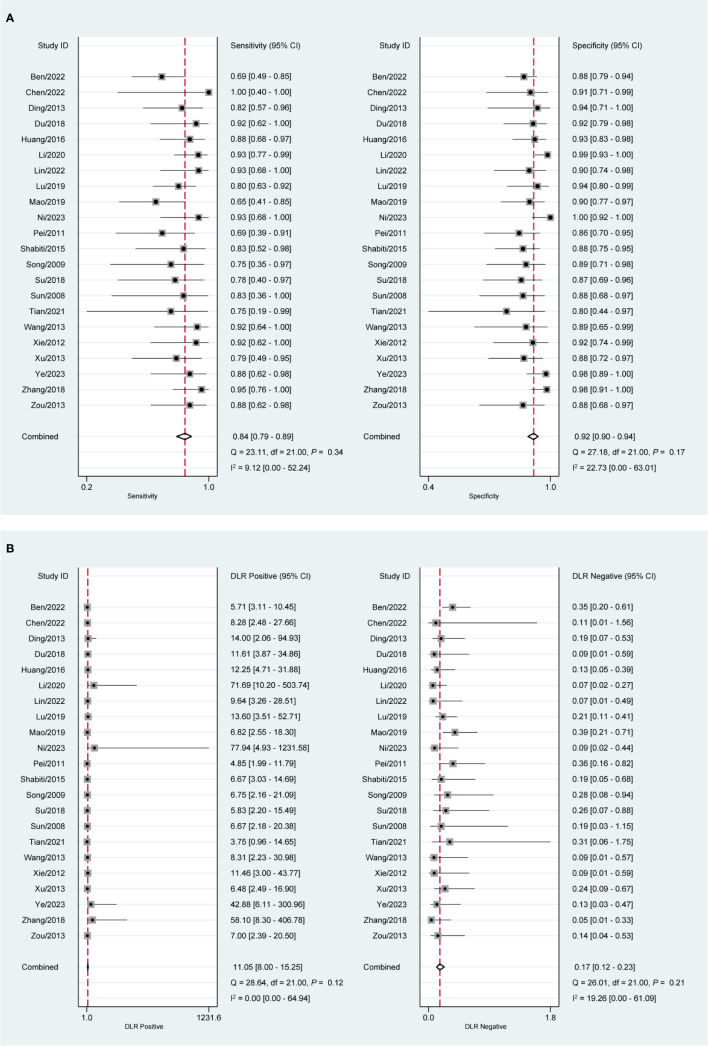
Forest plot of CEUS diagnosis of DMI in EC patients. **(A)** SEN and SPE; **(B)** PLR and NLR.

According to the plotted SROC curves (**Figure 2A**), the SROC curves showed a shoulder-arm distribution. The correlation coefficient was further calculated to be -0.54 (*P* = 0.01), indicating the presence of a threshold effect. **Figure 3A** depicts the combined SEN and SPE with values of 0.84 (95% CI: 0.79, 0.89) and 0.92 (95%CI: 0.90, 0.94). The values of PLR and NLR (**Figure 3B**) were 11.05 (95%CI: 8.00, 15.25) and 0.17 (95%CI: 0.12, 0.23). In terms of DOR (**Supplementary Figure S2A**) and AUC (**Figure 2A**), the values were calculated to be 64.90 (95%CI: 37.11, 113.51), and 0.95 (95%CI: 0.93, 0.97).

### Diagnostic value of CEUS in early EC with SMI

**Figure 4:**
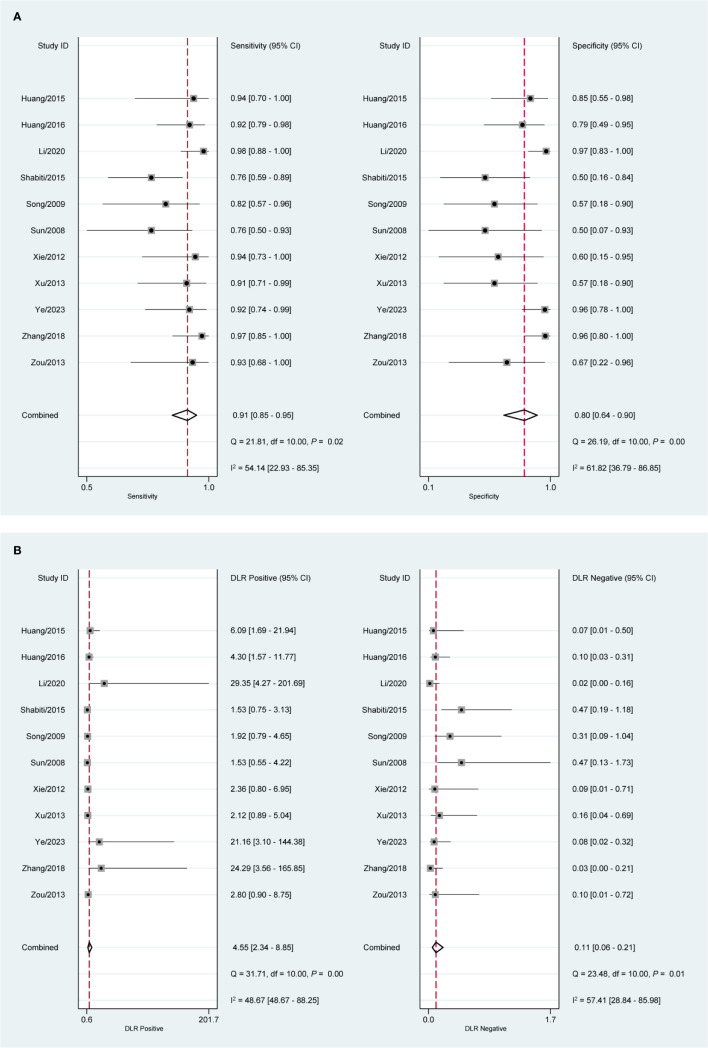
Forest plot of CEUS diagnosis of SMI in EC patients. **(A)** SEN and SPE; **(B)** PLR and NLR.

**Figure 2B** indicates a shoulder-arm distribution with a value of *P <*0.01. The combined SEN (**Figure 4A**) was 0.91 (95%CI: 0.85, 0.95) and the SPE was 0.80 (95%CI: 0.64, 0.90). The values of PLR, NLR, DOR, and AUC (**Figure 4B**, **Supplementary Figure S2B**, and **Figure 2B**) were 4.55 (95%CI: 2.34, 8.85), 0.11 (95%CI: 0.06, 0.21), 41.40 (95%CI: 12.14, 141.13), and 0.94 (95%CI: 0.91, 0.95).

### Subgroup analysis

**Table 2** summarizes the subgroup analysis based on ultrasound modality and invasive approach. The DOR and AUC were used to evaluate the diagnostic performance of different subgroups. DOR represents the ratio of the odds of a positive test result in diseased individuals to the odds in non-diseased individuals, with higher values indicating better discriminatory ability. AUC measures the overall diagnostic accuracy, where values closer to 1.0 indicate superior performance (51).

**Table 2:** Diagnostic efficacy of CEUS in EC with myometrial infiltration.

**Outcome**	**SEN**	**SPE**	**PLR**	**NLR**	**DOR**	**AUC**	**P-values of threshold**	**P-values of publication bias**
**DMI**	0.84 (0.79, 0.89)	0.92 (0.90, 0.94)	11.05 (8.00, 15.25)	0.17 (0.12, 0.23)	64.90 (37.11, 113.51)	0.95 (0.93, 0.97)	0.01	0.19
Transvaginal	0.83 (0.77, 0.88)	0.92 (0.89, 0.94)	10.22 (7.25, 14.41)	0.18 (0.13, 0.26)	55.97 (30.58, 102.44)	0.95 (0.92, 0.96)	0.10	
Color Doppler	0.85 (0.79, 0.90)	0.93 (0.90, 0.95)	11.69 (8.18, 16.71)	0.16 (0.11, 0.23)	74.31 (39.30, 140.50)	0.96 (0.93, 0.97)	0.02	
**SMI**	0.91 (0.85, 0.95)	0.80 (0.64, 0.90)	4.55 (2.34, 8.85)	0.11 (0.06, 0.21)	41.40 (12.14, 141.13)	0.94 (0.91, 0.95)	<0.01	<0.01
Transvaginal	0.93 (0.87, 0.96)	0.83 (0.67, 0.92)	5.53 (2.59, 11.82)	0.09 (0.05, 0.17)	62.65 (17.55, 223.68)	0.95 (0.93, 0.97)	0.06	
Color Doppler	0.92 (0.86, 0.96)	0.82 (0.66, 0.91)	5.02 (2.45, 10.28)	0.10 (0.05, 0.20)	50.24 (13.62, 185.30)	0.94 (0.92, 0.96)	0.01	

CEUS, Contrast-enhanced ultrasound; EC, Endometrial cancer; DMI, Deep myometrial infiltration; SMI, Superficial myometrial invasion; SEN, Sensitivity; SPE, Specificity; PLR, Positive likelihood ratio; NLR, Negative likelihood ratio; DOR, Diagnostic odds ratio; AUC, Area under curve.

Regarding ultrasound modality, color Doppler imaging showed improved diagnostic efficacy compared to greyscale ultrasound. The color Doppler subgroup demonstrated higher SEN, SPE, and AUC, suggesting that Doppler-based CEUS provides enhanced diagnostic accuracy for detecting myometrial invasion (52). The pooled DOR for the color Doppler group was 74.32 (95% CI: 39.31–140.52), indicating a significantly higher likelihood of correctly distinguishing between patients with and without disease (**Supplementary Figure S3A**, **Supplementary Figure S4**).

For the invasive approach, transvaginal CEUS exhibited no threshold effect when diagnosing both DMI and SMI (**Supplementary Figure S3B**, **Supplementary Figure S5**, P > 0.05). This consistency suggests that transvaginal CEUS provides stable diagnostic accuracy across different studies, likely due to its higher spatial resolution and direct visualization of myometrial invasion (53). In the diagnosis of DMI, transvaginal CEUS showed a SEN of 0.83 (95% CI: 0.77–0.88), SPE of 0.92 (95% CI: 0.89–0.94), and AUC of 0.95 (95% CI: 0.92–0.96). Similarly, for SMI, both color Doppler (**Supplementary Figure S6**) and transvaginal CEUS (**Supplementary Figure S7**) demonstrated superior diagnostic performance, with DOR values of 50.24 (95% CI: 13.62–185.30) and 62.65 (95% CI: 17.55–223.68), respectively. These findings highlight transvaginal CEUS as an optimal imaging technique for detecting myometrial invasion with enhanced sensitivity and specificity.

When examining the diagnostic efficacy of CEUS for SMI (**Supplementary Figure S3**), there was no threshold effect in the transvaginal group (*P >*0.05). In addition, the combined SEN, SPE, and AUC of the diagnostic tests were improved in both color Doppler (**Supplementary Figure S6**) and transvaginal (**Supplementary Figure S7**) groups, with DOR values of 50.24 (95% CI: 13.62, 185.30) and 62.65 (95% CI: 17.55, 223.68), respectively.

### Detection of the publication bias

**Figure 5:**
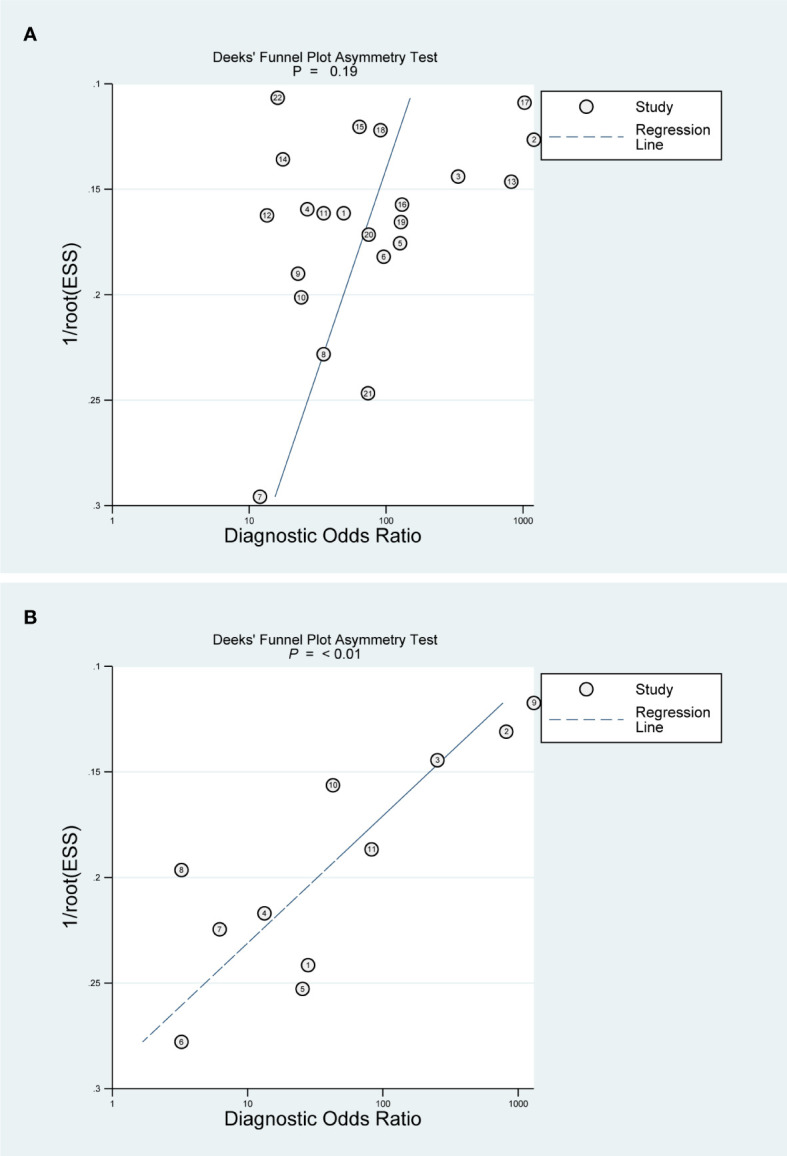
Deeks’ funnel plot asymmetry test for publication bias. **(A)** DMI; **(B)** SMI.

The assessments of publication bias were performed for DMI (**Figure 5A**) and SMI (**Figure 5B**). Deeks’ funnel plots reveal a publication bias for the diagnosis of SMI in EC patients using CEUS.

## Discussion

CEUS is a technique for ultrasound imaging utilizing an ultrasound contrast agent, usually a suspension of air bubbles (8). These tiny bubbles (usually formed by inert gases encased in a phospholipid shell) oscillate or burst when they encounter ultrasound waves as they travel through the bloodstream, producing a strong reflected signal (54). By scrutinizing these enhanced signals, physicians can obtain clearer tissue imaging and more detailed blood flow information. CEUS is considered a non-invasive diagnostic method that enhances the contrast between lesions and normal tissues and improves diagnostic accuracy. Furthermore, contrast agents do not raise the load of renal excretion, and CEUS is less expensive than other imaging modalities (55). Due to these advantages, CEUS technology has a wide range of applications in many clinical fields, especially in the diagnosis of liver, kidney, and blood vessels (56–58). Recently, the powerful efficiency demonstrated by CEUS in the diagnosis of EC has also attracted our attention. In this study, a total of 23 papers were included and 1247 EC patients were recruited to participate in the study. The results revealed that CEUS had a high value in the diagnosis of EC. For both DMI and SMI, SEN and SPE exceeded 0.8, while the AUC curves surpassed 0.9. In addition, high PLR and low NLR indicated that the diagnostic tests performed well in correctly identifying the true disease state.

One of the key strengths of this study is its comprehensive methodology, including a large sample size and systematic analysis of multiple databases. By incorporating data from various clinical settings, this study enhances the generalizability of its findings. Additionally, the subgroup analyses provided insights into the diagnostic performance of CEUS under different conditions, such as transvaginal versus transabdominal approaches and different ultrasound modalities.

However, several limitations should be acknowledged. First, study heterogeneity may introduce potential biases, particularly due to variations in imaging protocols, operator experience, and ultrasound equipment. Second, publication bias remains a concern, as studies with negative or less favorable results may be underreported. Third, the presence of a threshold effect in certain analyses suggests variability in diagnostic cutoffs, which could affect test performance. Lastly, while CEUS demonstrated high diagnostic accuracy, it still falls short of the histopathological gold standard (final paraffin-embedded pathology) (59). Therefore, future studies should aim to standardize imaging protocols, improve reporting transparency, and validate findings in larger, multicenter cohorts.

Given the comparable diagnostic performance of CEUS and MRI in assessing myometrial invasion, CEUS presents a practical alternative in resource-limited settings where MRI or CT may be unavailable or cost-prohibitive. Unlike MRI, CEUS is more accessible, cost-effective, and has fewer contraindications, making it a viable first-line imaging option for preoperative staging of EC. Additionally, CEUS may serve as a complementary tool to MRI, especially in cases where MRI findings are inconclusive or when contrast-enhanced MRI is contraindicated. Future studies should explore integrating CEUS into existing diagnostic pathways and assessing its role in risk stratification algorithms for myometrial infiltration.

Our findings align with prior meta-analyses assessing TVS and MRI for myometrial invasion in EC. A previous meta-analysis reported that MRI exhibited higher sensitivity than TVS, but the difference was not statistically significant (60, 61). CEUS, as an evolving imaging modality, has demonstrated improved diagnostic efficiency over conventional ultrasound, with DOR and AUC values comparable to MRI (62, 63). A recent retrospective study comparing CT, MRI, intraoperative frozen section (IFS), and final histopathology found that CEUS provided diagnostic accuracy close to MRI while being less invasive and more cost-effective (64). Additionally, a meta-analysis evaluating dynamic contrast-enhanced MRI (DCE-MRI) for detecting cervical infiltration in EC reported diagnostic accuracy metrics similar to those of CEUS (65). These comparisons reinforce the clinical value of CEUS in non-invasively assessing myometrial invasion while acknowledging its limitations relative to histopathology (66).

Beyond imaging-based methods, other diagnostic tools and algorithms have been explored for assessing myometrial invasion in EC. Recent studies suggest that inflammatory markers, such as the neutrophil-to-lymphocyte ratio (67) and SIR-En (68), correlate with the presence of myometrial infiltration. Additionally, biomarkers like the monocyte-to-lymphocyte ratio and platelet-to-lymphocyte ratio (69, 70) have shown promise as non-invasive and cost-effective diagnostic indicators. Given their high accuracy, these biomarkers could be integrated with CEUS to enhance diagnostic precision. Future research should focus on developing multimodal diagnostic approaches that combine CEUS with biomarker-based risk stratification to improve early detection and clinical decision-making in EC.

## Conclusion and future perspective

In conclusion, our study demonstrates that CEUS is a valuable diagnostic modality for assessing myometrial and serosal infiltration in EC. The high sensitivity, specificity, and AUC values observed indicate that CEUS provides reliable diagnostic performance comparable to existing imaging techniques, with additional benefits in terms of cost-effectiveness, accessibility, and safety. Given its advantages over MRI and CT, CEUS has the potential to be integrated into preoperative staging protocols to aid clinical decision-making.

Despite these strengths, certain limitations must be acknowledged, including study heterogeneity, potential operator-dependent variability, and the need for standardized imaging protocols. Additionally, the presence of publication bias suggests that further large-scale, multicenter studies with standardized methodologies are warranted.

Moving forward, research should focus on standardizing CEUS imaging protocols to improve reproducibility and diagnostic consistency across different clinical settings. Further investigations should evaluate the utility of CEUS in advanced-stage EC, particularly in assessing lymph node involvement and distant metastases, where its role remains unclear. Additionally, artificial intelligence (AI)-assisted image analysis may enhance the diagnostic accuracy of CEUS by reducing inter-operator variability. Future studies should also explore the integration of CEUS with existing risk-stratification algorithms, such as those referenced in recent literature, to optimize individualized treatment planning.

By addressing these research gaps, CEUS could further solidify its role as a non-invasive, efficient, and accessible diagnostic tool for the management of endometrial cancer.

## Data Availability

The original contributions presented in the study are included in the article/**Supplementary Material**. Further inquiries can be directed to the corresponding author.

## Supplementary material

Supplementary Figure 1 The quality assessment of eligible studies. **(A)** Risk of bias summary; **(B)** Risk of bias graph.

Supplementary Figure 2 Forest plot of DOR for diagnosis of EC myometrial infiltration using CEUS. **(A)** DMI; **(B)** SMI.

Supplementary Figure 3 SROC curve of subgroup analysis. **(A)** Diagnosis of DMI using CEUS in the color Doppler group; **(B)** Diagnosis of DMI using CEUS in the transvaginal group; **(C)** Diagnosis of SMI using CEUS in the color Doppler group; **(D)** Diagnosis of SMI using CEUS in the transvaginal group.

Supplementary Figure 4 Forest plot of DMI diagnosis using CEUS in the color Doppler group. **(A)** SEN and SPE; **(B)** PLR and NLR; **(C)** DOR.

Supplementary Figure 5 Forest plot of DMI diagnosis using CEUS in the transvaginal group. **(A)** SEN and SPE; **(B)** PLR and NLR; **(C)** DOR.

Supplementary Figure 6 Forest plot of SMI diagnosis using CEUS in the color Doppler group. **(A)** SEN and SPE; **(B)** PLR and NLR; **(C)** DOR.

Supplementary Figure 7 Forest plot of SMI diagnosis using CEUS in the transvaginal group. **(A)** SEN and SPE; **(B)** PLR and NLR; **(C)** DOR.
