# Supplementary figures and images for: Diagnostic value of contrast-enhanced ultrasound for the depth of myometrial infiltration in early endometrial cancer: a meta-analysis
Source: Front Oncol. 2025 Mar 7;15:1493246. doi: 10.3389/fonc.2025.1493246 (PMC11921045; doi:10.3389/fonc.2025.1493246)

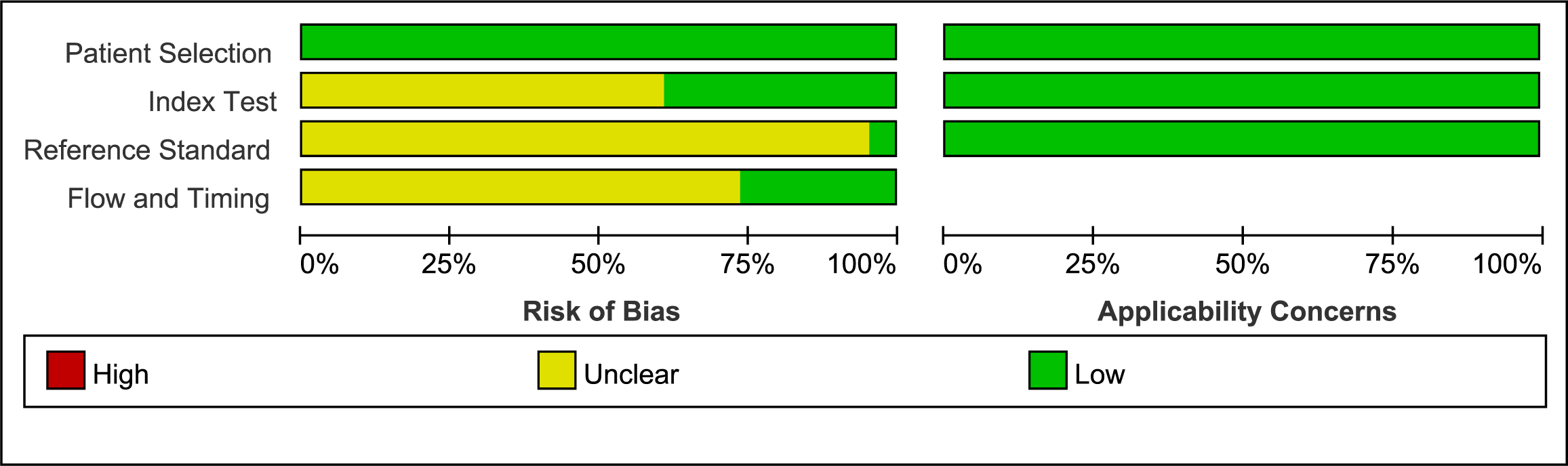

Supplement: Supplementary Figure 1 — The quality assessment of eligible studies. (A) Risk of bias summary; (B) Risk of bias graph. [file DataSheet1.zip › Figure S1A.tif]

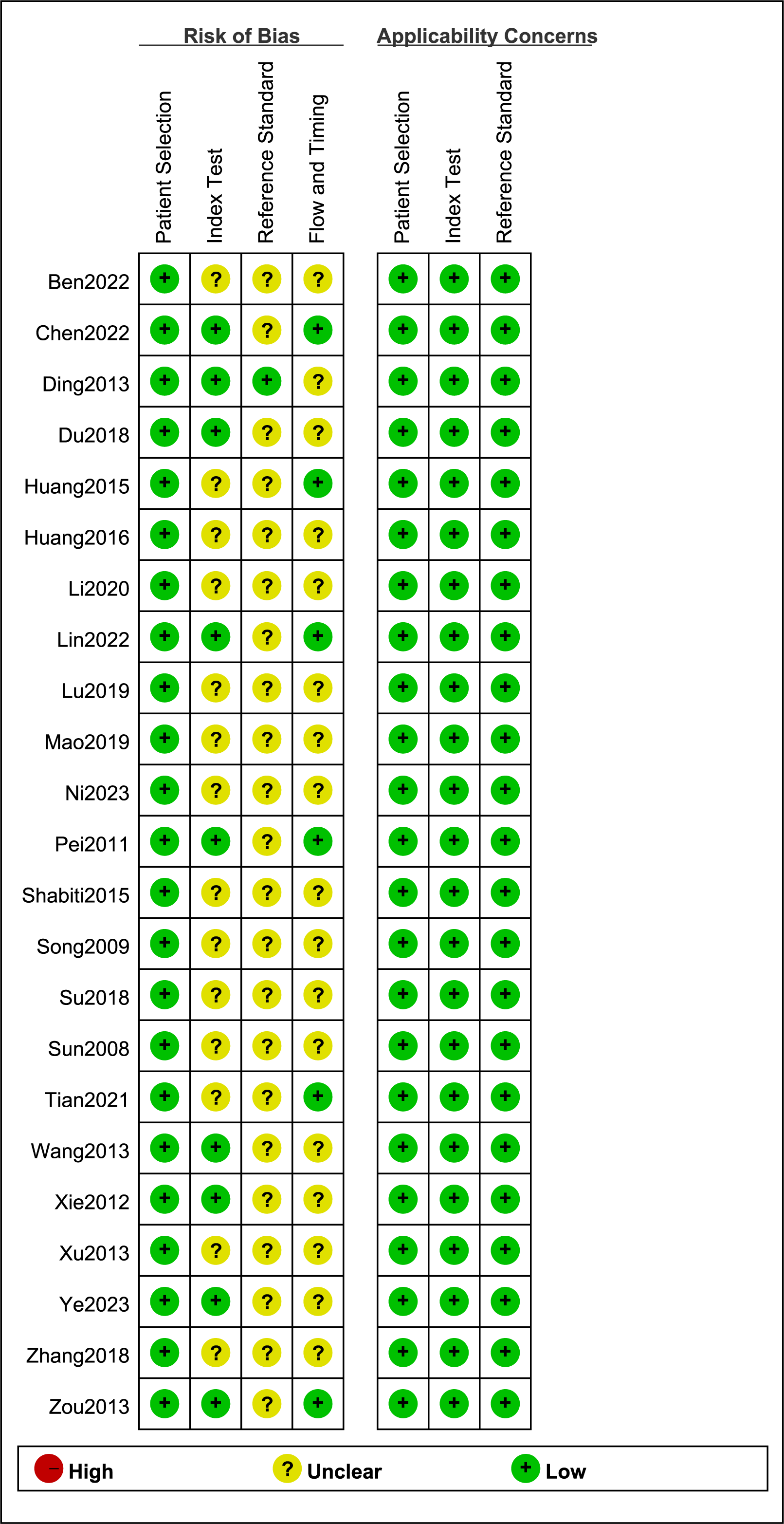

Supplement: Supplementary Figure 1 — The quality assessment of eligible studies. (A) Risk of bias summary; (B) Risk of bias graph. [file DataSheet1.zip › Figure S1B.tif]

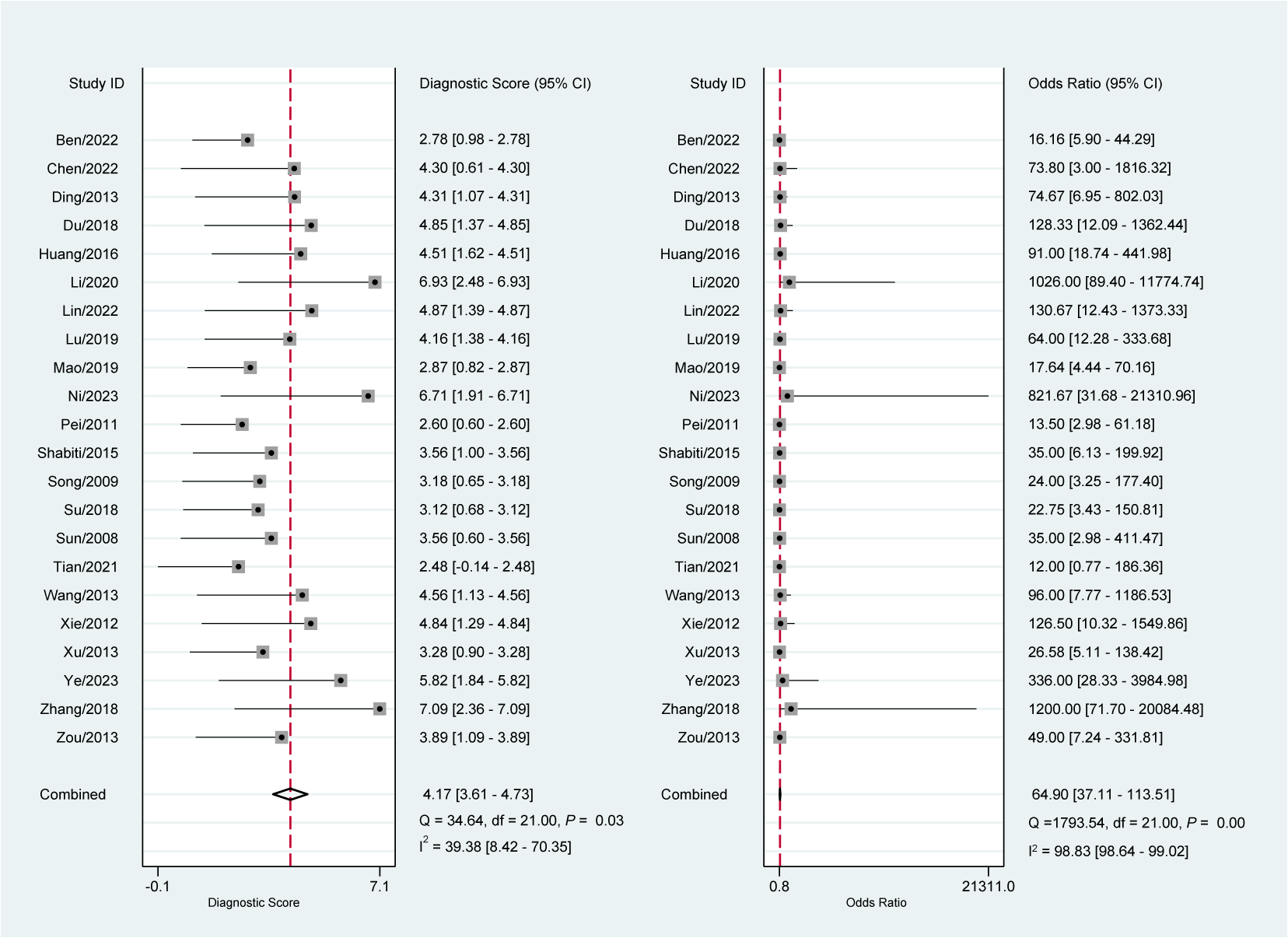

Supplement: Supplementary Figure 1 — The quality assessment of eligible studies. (A) Risk of bias summary; (B) Risk of bias graph. [file DataSheet1.zip › Figure S2A.tif]

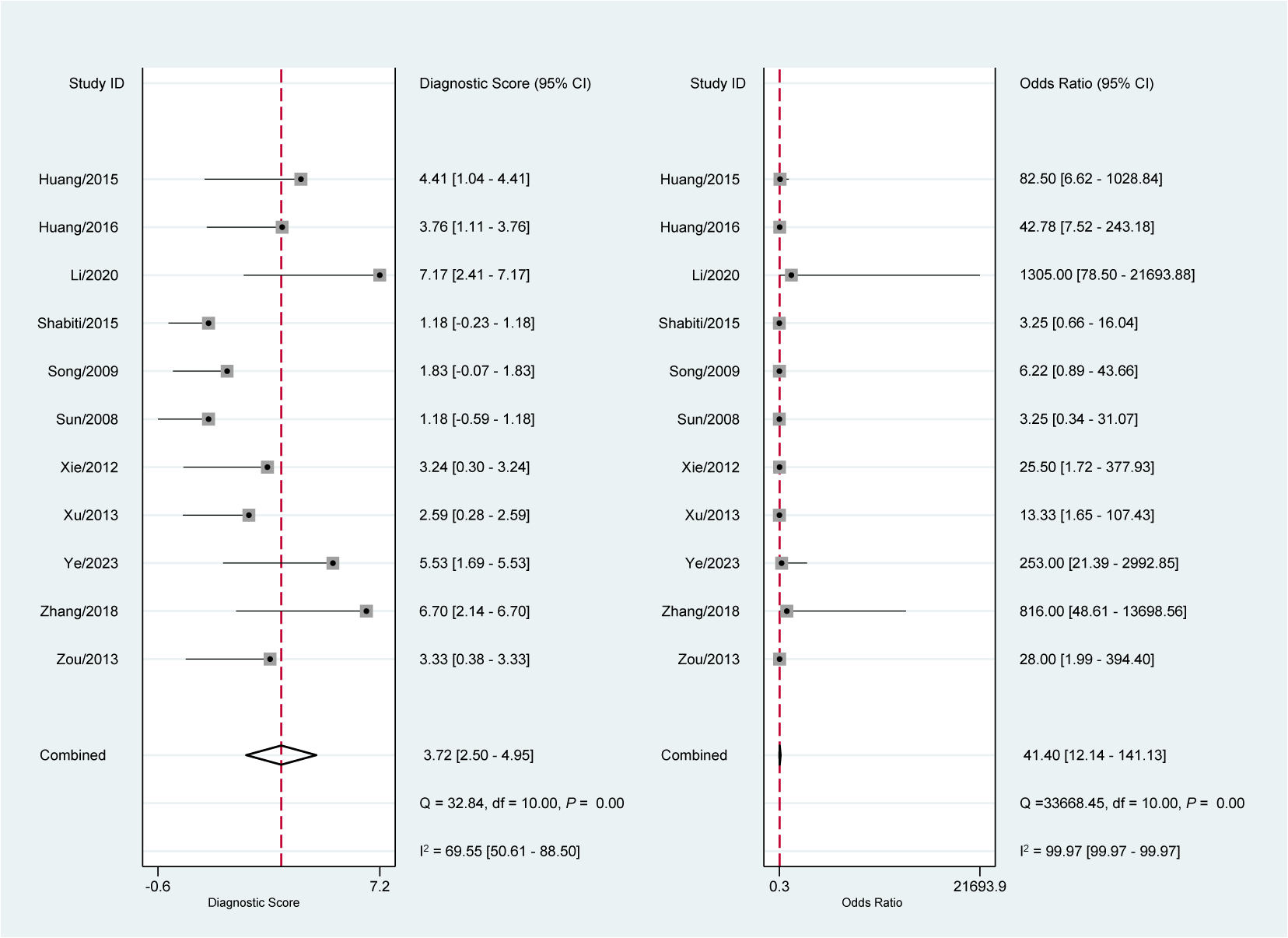

Supplement: Supplementary Figure 1 — The quality assessment of eligible studies. (A) Risk of bias summary; (B) Risk of bias graph. [file DataSheet1.zip › Figure S2B.tif]

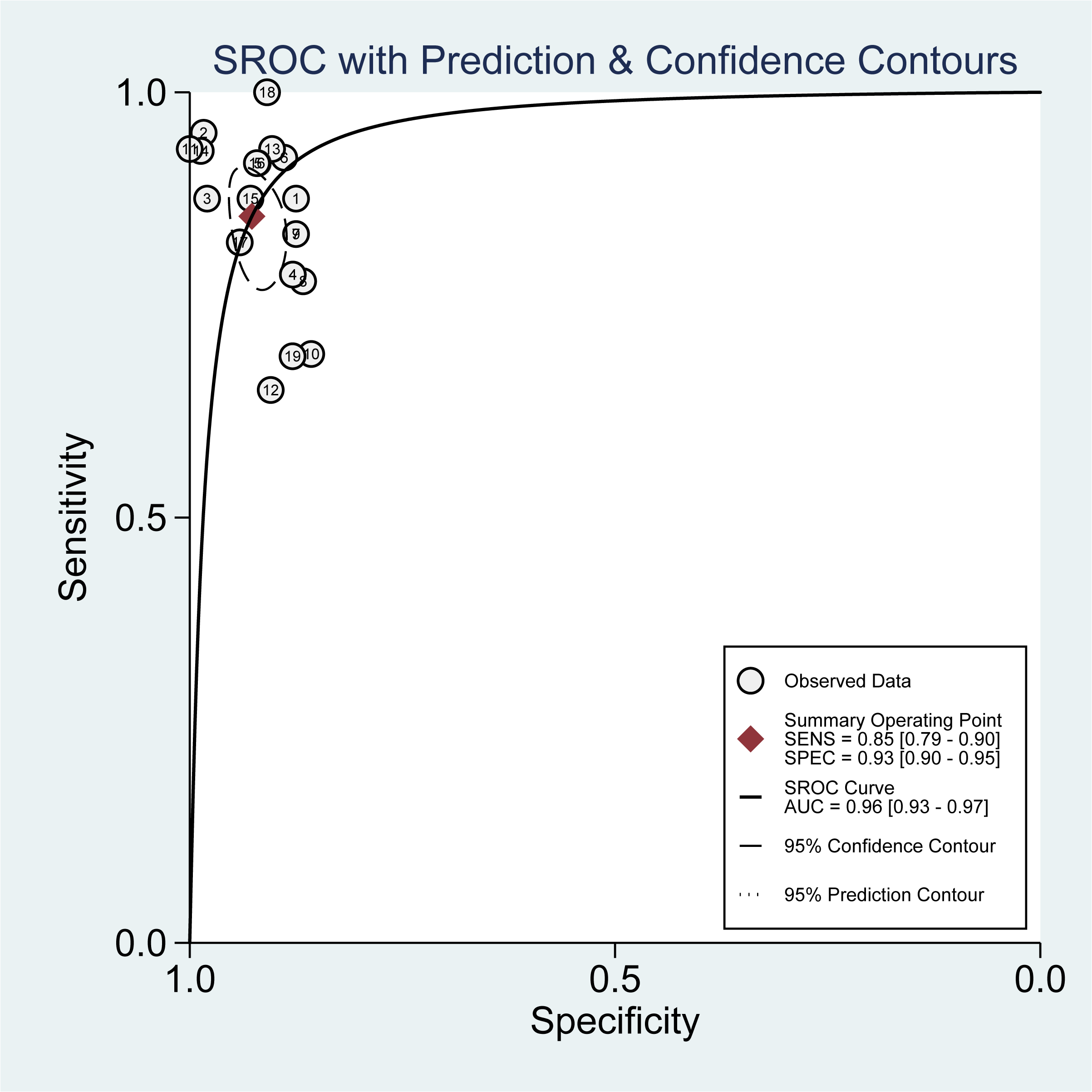

Supplement: Supplementary Figure 1 — The quality assessment of eligible studies. (A) Risk of bias summary; (B) Risk of bias graph. [file DataSheet1.zip › Figure S3A.tif]

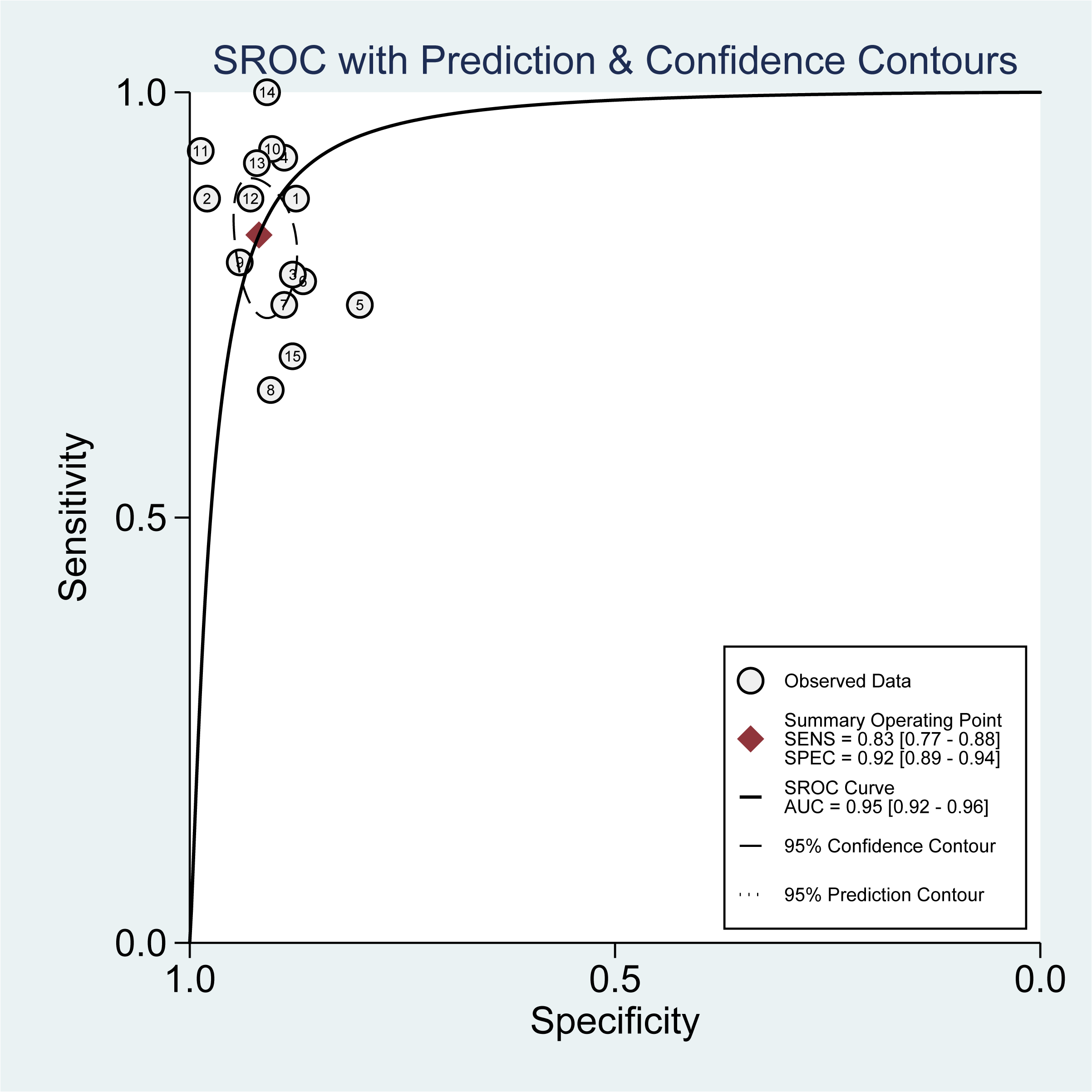

Supplement: Supplementary Figure 1 — The quality assessment of eligible studies. (A) Risk of bias summary; (B) Risk of bias graph. [file DataSheet1.zip › Figure S3B.tif]

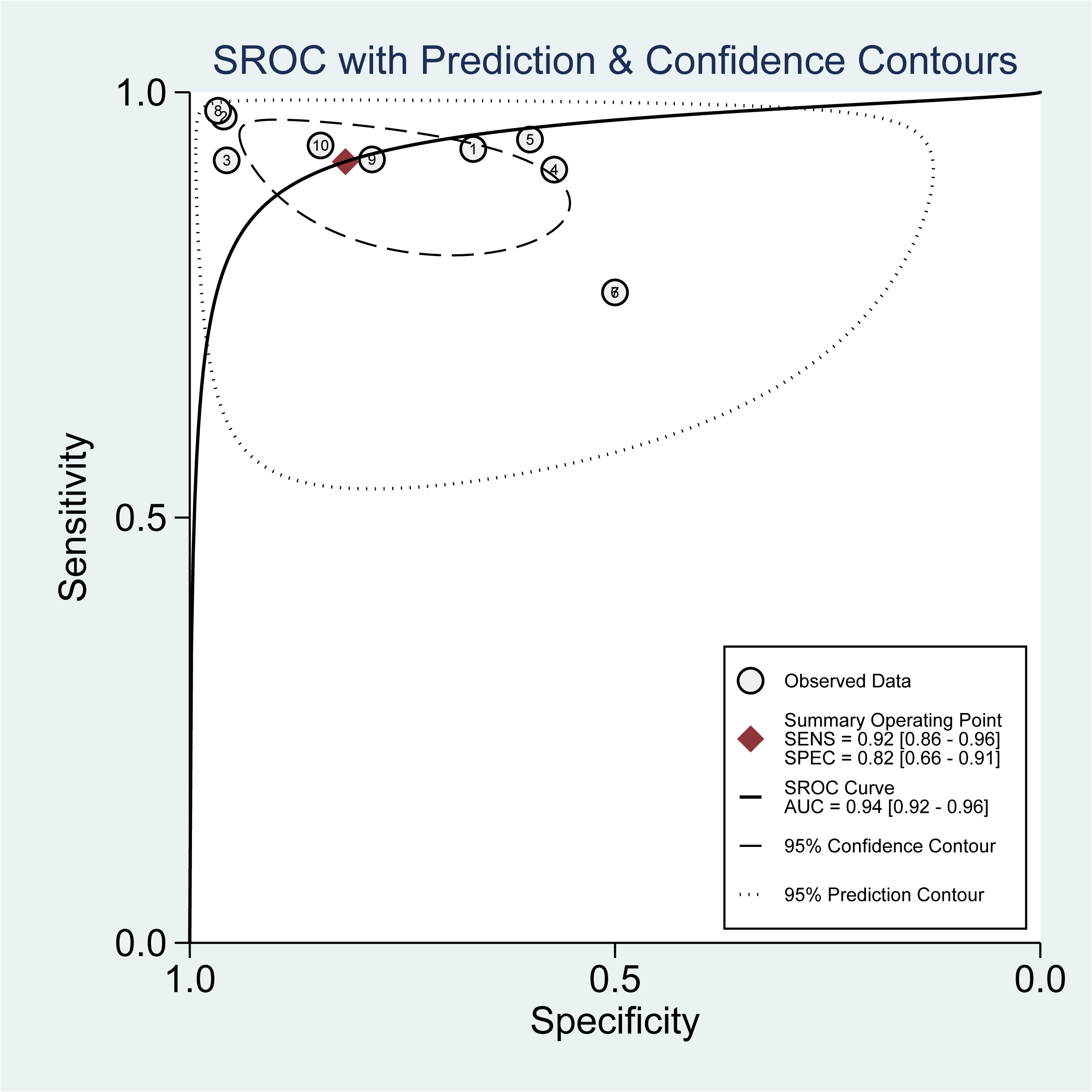

Supplement: Supplementary Figure 1 — The quality assessment of eligible studies. (A) Risk of bias summary; (B) Risk of bias graph. [file DataSheet1.zip › Figure S3C.tif]

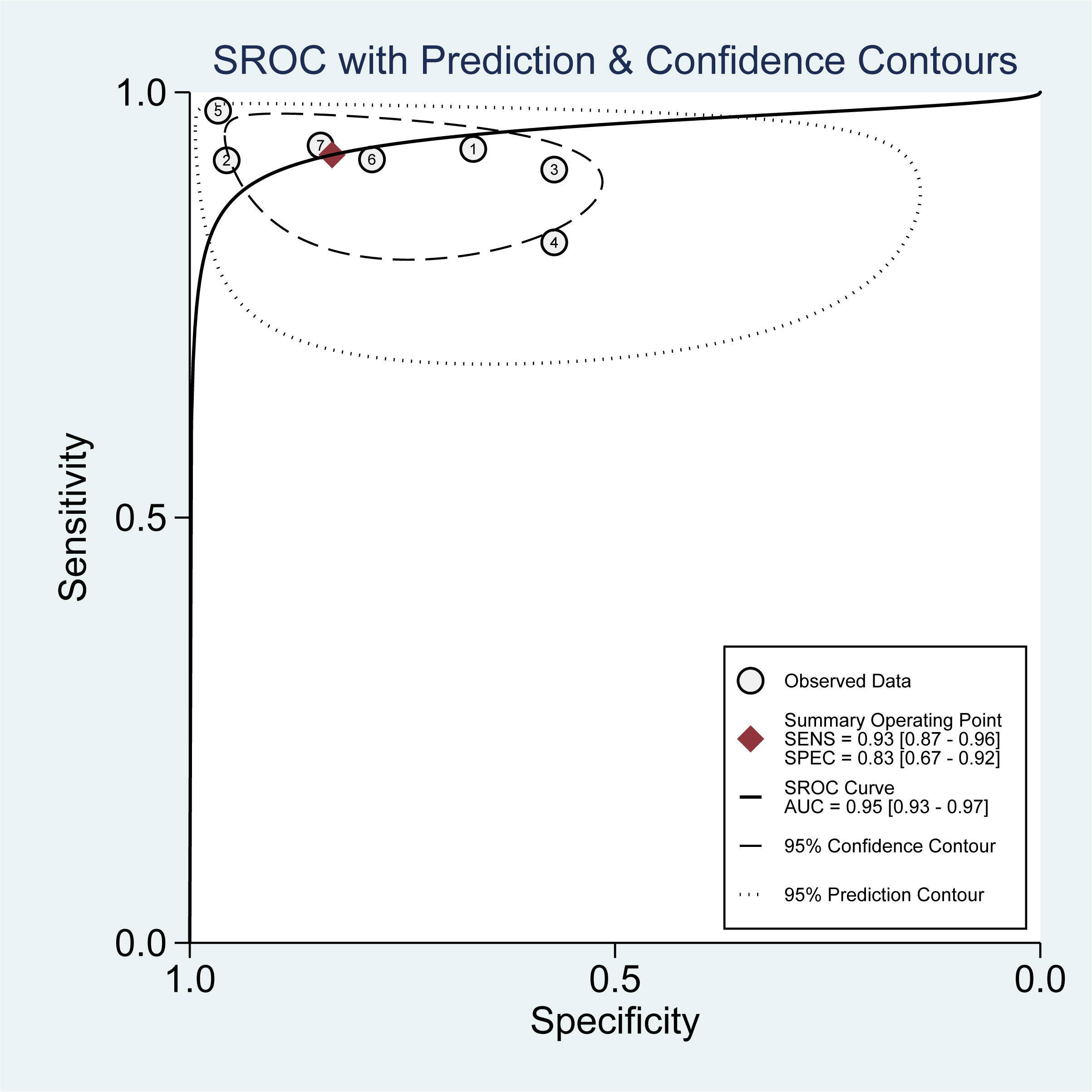

Supplement: Supplementary Figure 1 — The quality assessment of eligible studies. (A) Risk of bias summary; (B) Risk of bias graph. [file DataSheet1.zip › Figure S3D.tif]

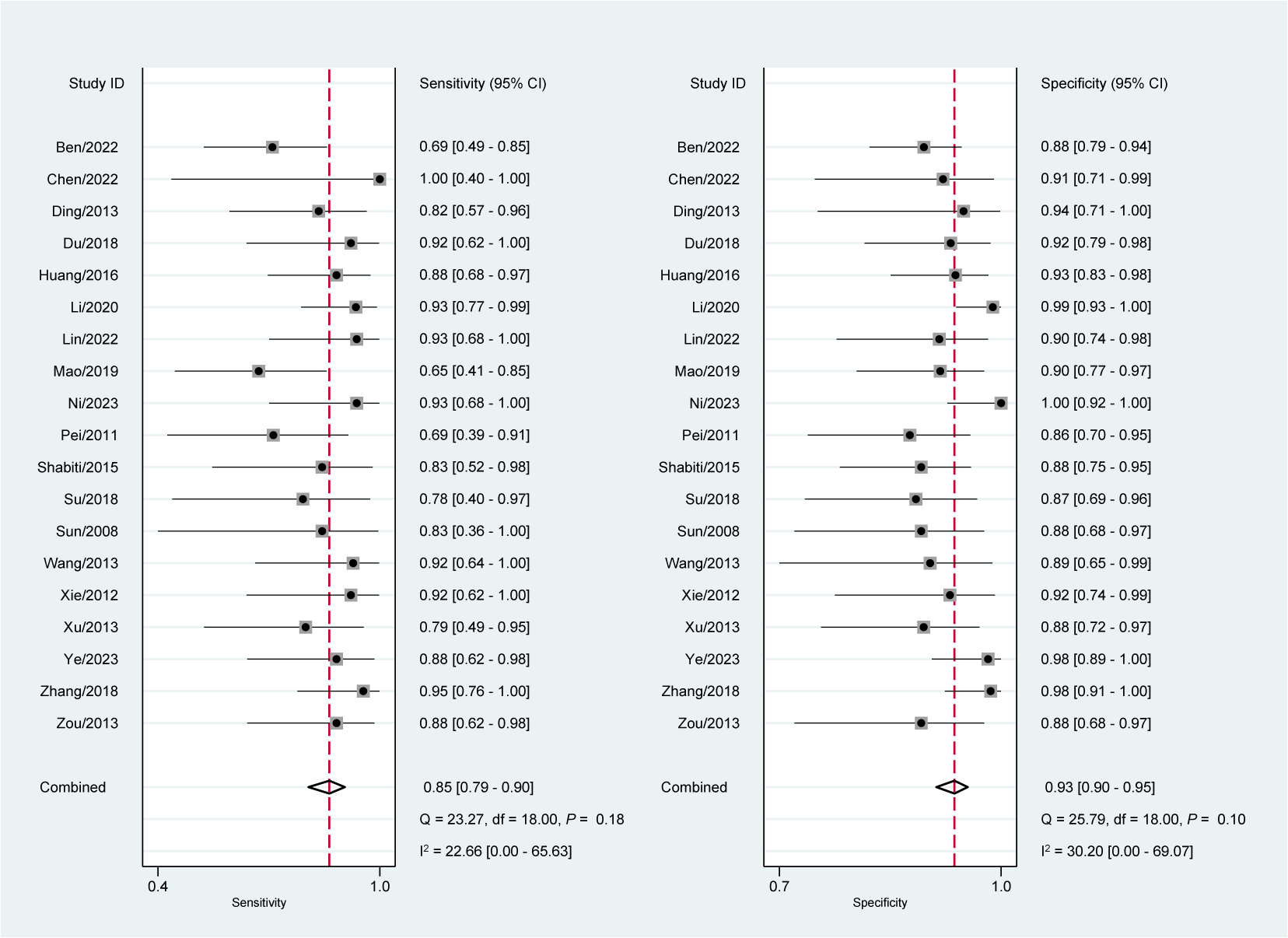

Supplement: Supplementary Figure 1 — The quality assessment of eligible studies. (A) Risk of bias summary; (B) Risk of bias graph. [file DataSheet1.zip › Figure S4A.tif]

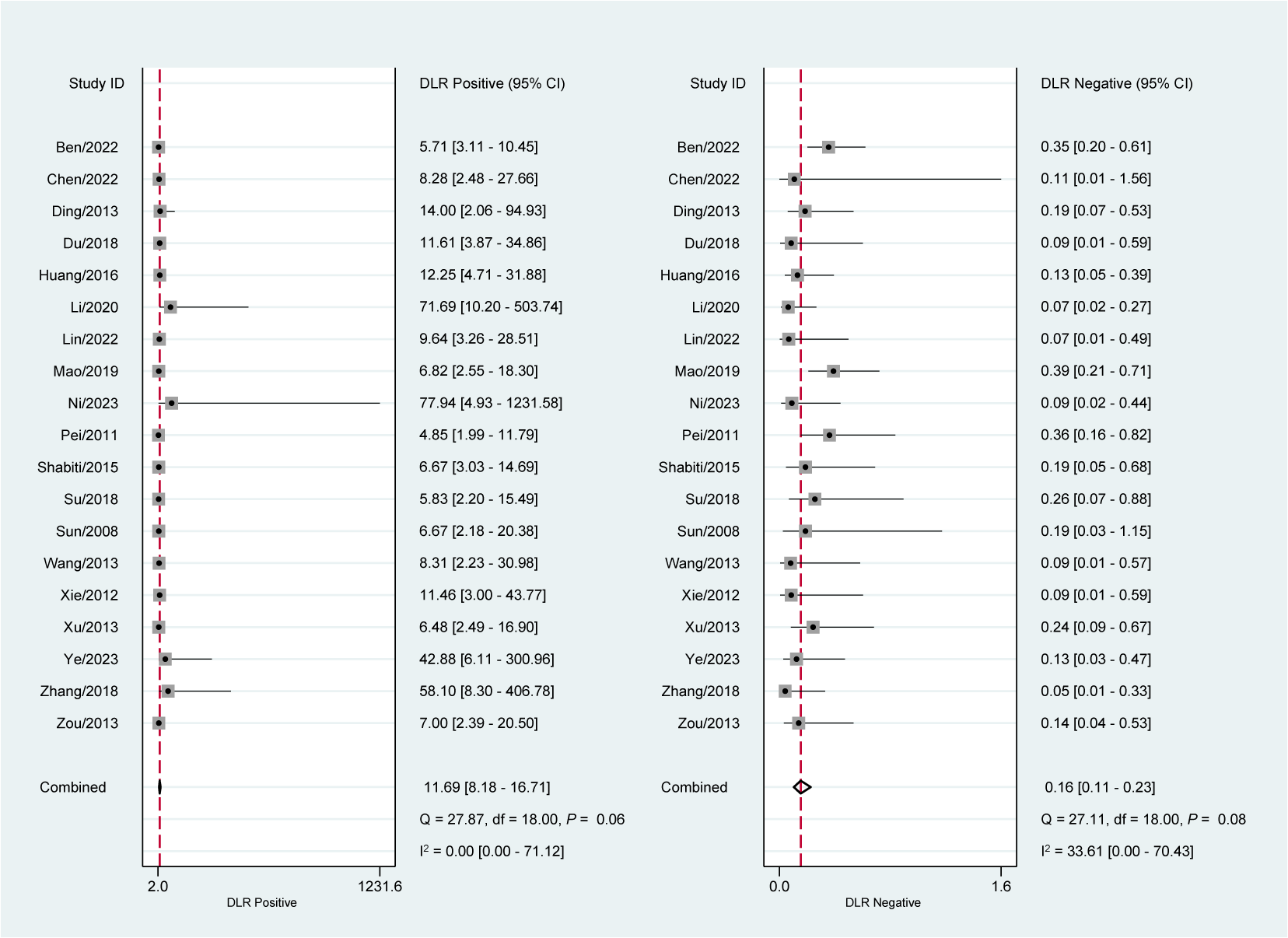

Supplement: Supplementary Figure 1 — The quality assessment of eligible studies. (A) Risk of bias summary; (B) Risk of bias graph. [file DataSheet1.zip › Figure S4B.tif]

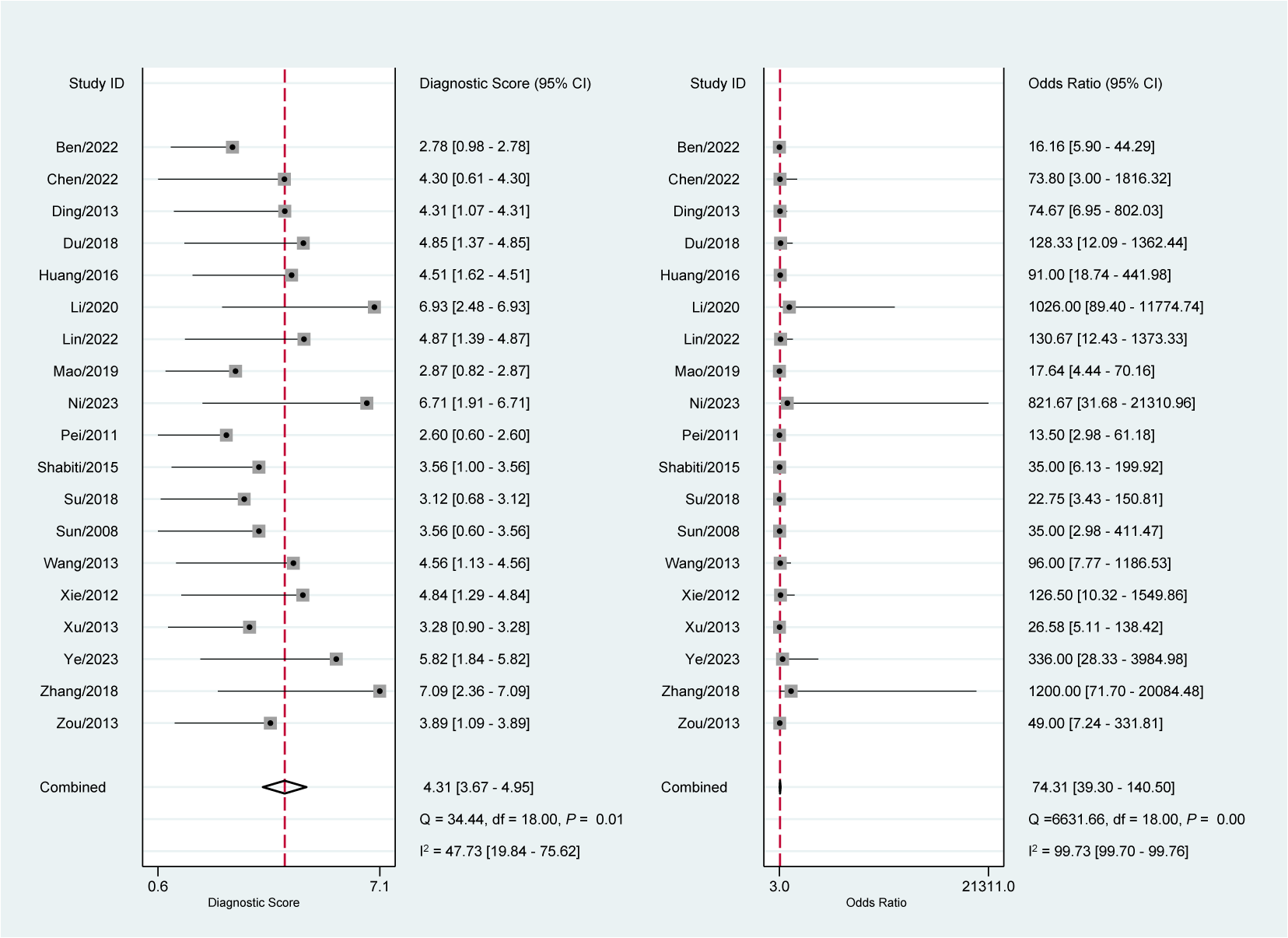

Supplement: Supplementary Figure 1 — The quality assessment of eligible studies. (A) Risk of bias summary; (B) Risk of bias graph. [file DataSheet1.zip › Figure S4C.tif]

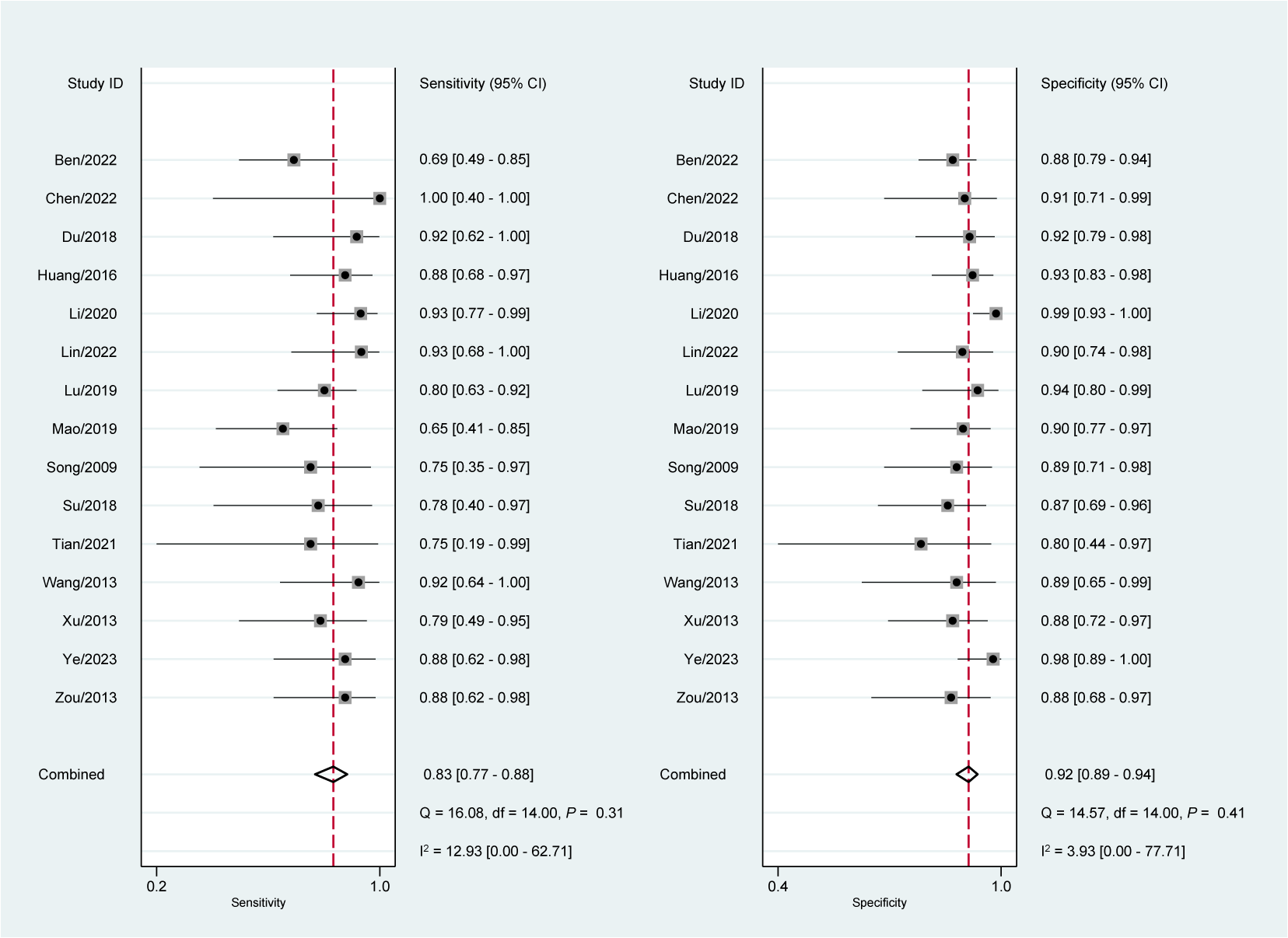

Supplement: Supplementary Figure 1 — The quality assessment of eligible studies. (A) Risk of bias summary; (B) Risk of bias graph. [file DataSheet1.zip › Figure S5A.tif]

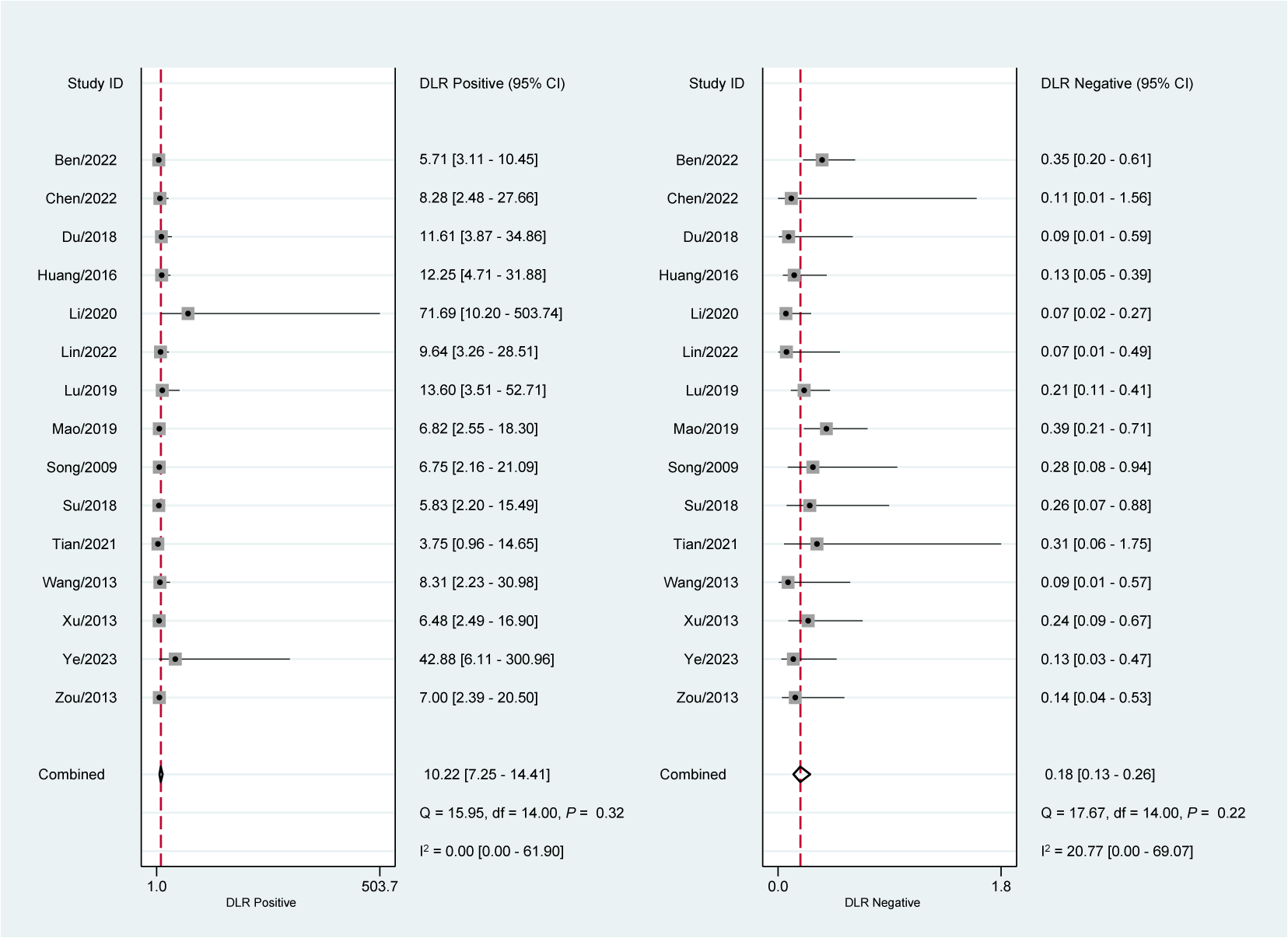

Supplement: Supplementary Figure 1 — The quality assessment of eligible studies. (A) Risk of bias summary; (B) Risk of bias graph. [file DataSheet1.zip › Figure S5B.tif]

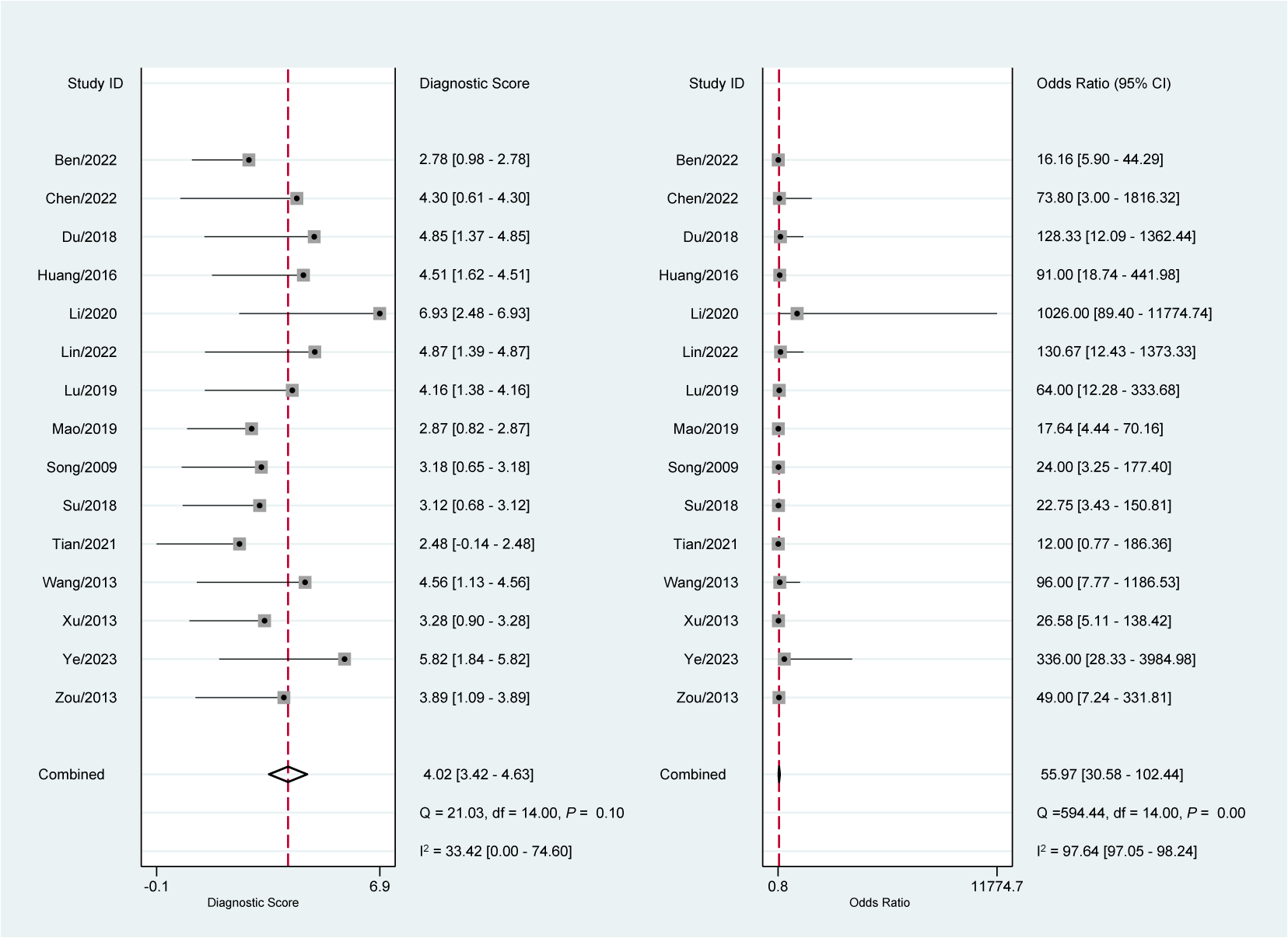

Supplement: Supplementary Figure 1 — The quality assessment of eligible studies. (A) Risk of bias summary; (B) Risk of bias graph. [file DataSheet1.zip › Figure S5C.tif]

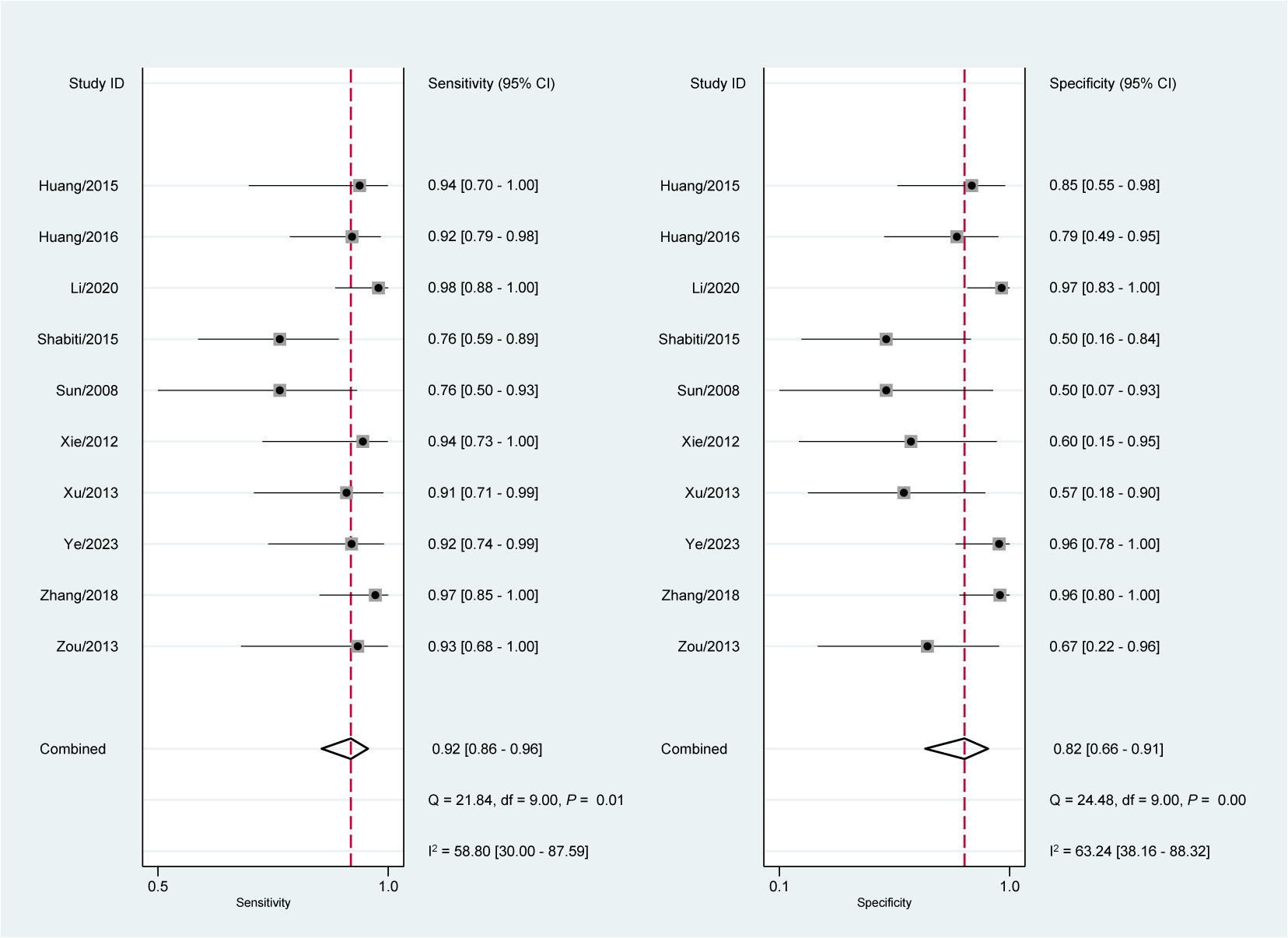

Supplement: Supplementary Figure 1 — The quality assessment of eligible studies. (A) Risk of bias summary; (B) Risk of bias graph. [file DataSheet1.zip › Figure S6A.tif]

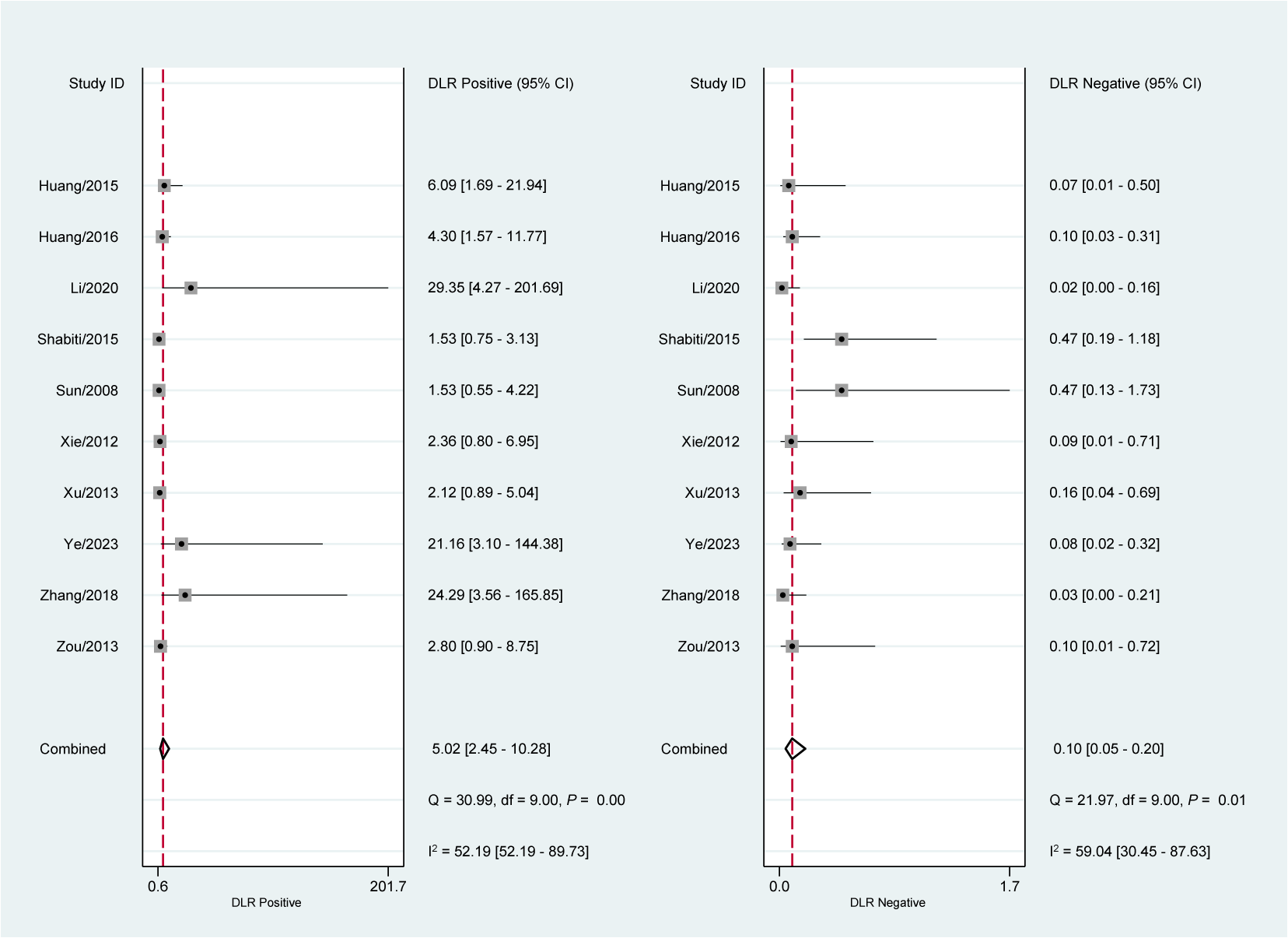

Supplement: Supplementary Figure 1 — The quality assessment of eligible studies. (A) Risk of bias summary; (B) Risk of bias graph. [file DataSheet1.zip › Figure S6B.tif]

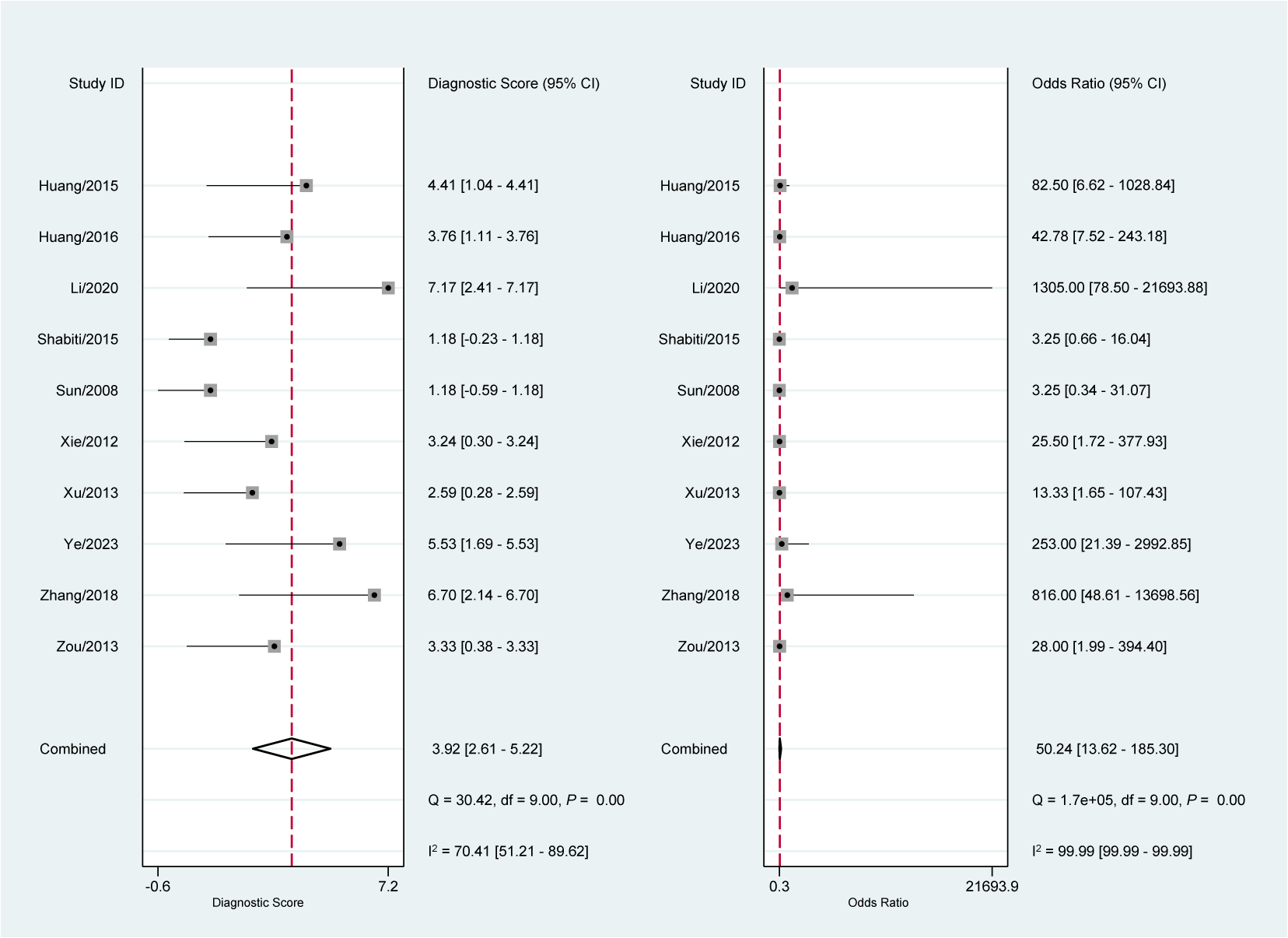

Supplement: Supplementary Figure 1 — The quality assessment of eligible studies. (A) Risk of bias summary; (B) Risk of bias graph. [file DataSheet1.zip › Figure S6C.tif]

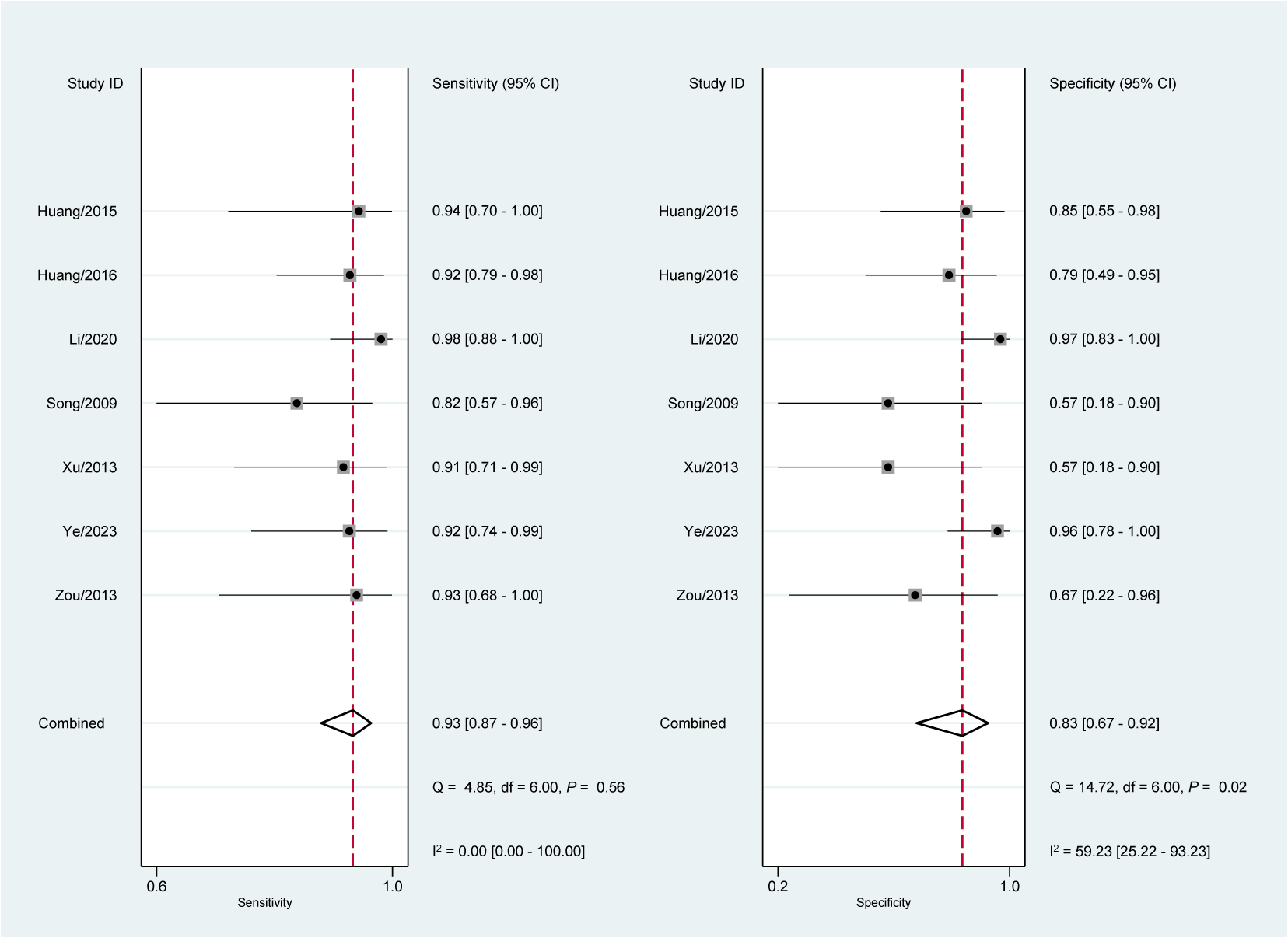

Supplement: Supplementary Figure 1 — The quality assessment of eligible studies. (A) Risk of bias summary; (B) Risk of bias graph. [file DataSheet1.zip › Figure S7A.tif]

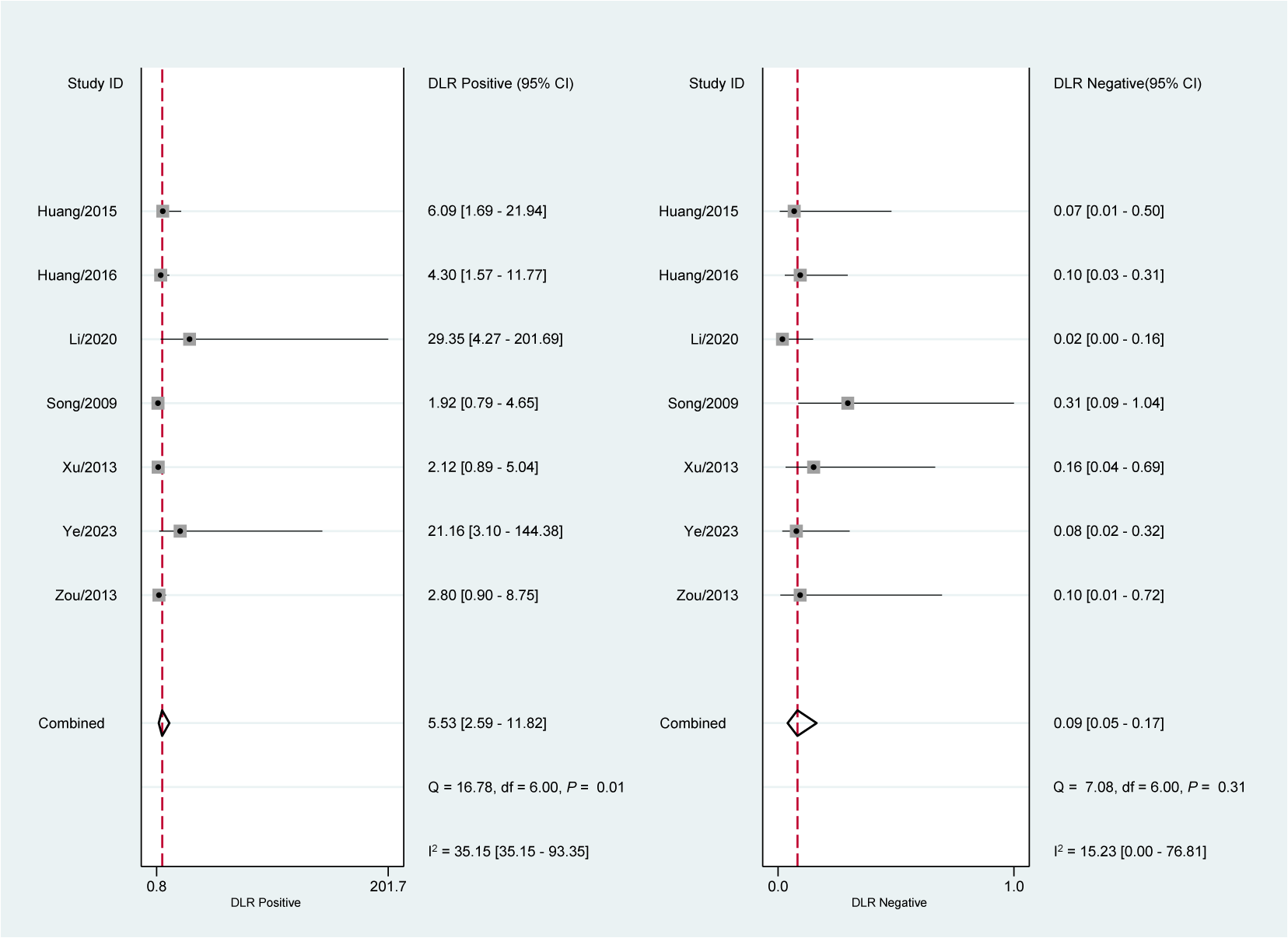

Supplement: Supplementary Figure 1 — The quality assessment of eligible studies. (A) Risk of bias summary; (B) Risk of bias graph. [file DataSheet1.zip › Figure S7B.tif]

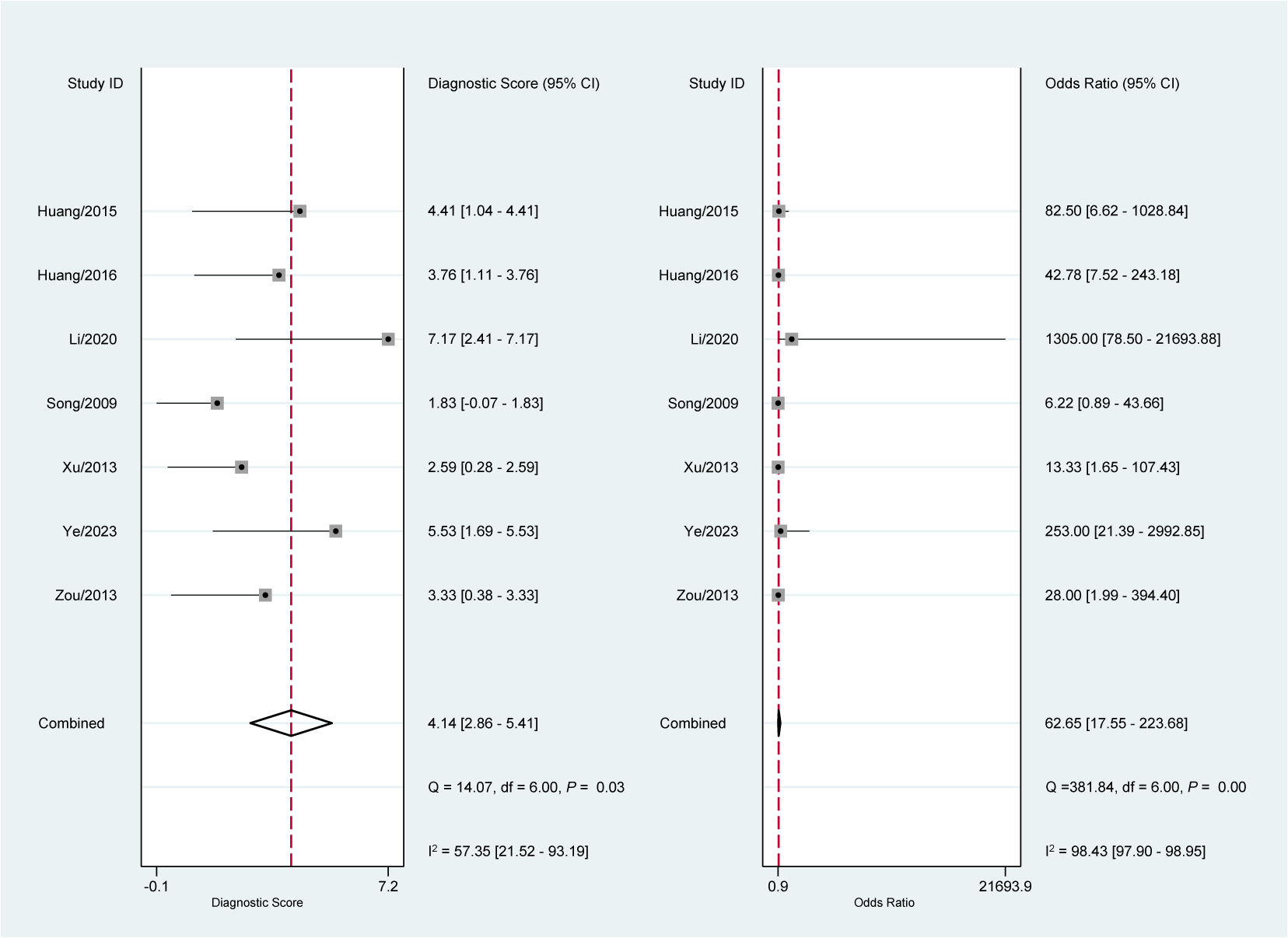

Supplement: Supplementary Figure 1 — The quality assessment of eligible studies. (A) Risk of bias summary; (B) Risk of bias graph. [file DataSheet1.zip › Figure S7C.tif]
