# Supplementary material for: Diagnostic value of contrast-enhanced ultrasound for the depth of myometrial infiltration in early endometrial cancer: a meta-analysis
Source: Front Oncol. 2025 Mar 7;15:1493246. doi: 10.3389/fonc.2025.1493246 (PMC11921045; doi:10.3389/fonc.2025.1493246)
Supplement: Supplementary file 2 [file DataSheet2.docx]

Table S1 The search formula of PubMed

| **Items** | **Content** |
| --- | --- |
| #1 | (((((((((((((Endometrial Neoplasms[Title/Abstract]) OR (Endometrial Neoplasm[Title/Abstract])) OR (Endometrial Carcinoma*[Title/Abstract])) OR (Endometrial Cancer*[Title/Abstract])) OR (Endometrium Cancer*[Title/Abstract])) OR (Carcinoma of Endometrium[Title/Abstract])) OR (Endometrium Carcinoma*[Title/Abstract])) OR (Cancer of Endometrium[Title/Abstract])) OR (Endometrium Cancer*[Title/Abstract])) OR (Carcinoma, Endometrioid[Title/Abstract])) OR (Carcinomas, Endometrioid[Title/Abstract])) OR (Endometrioid Carcinoma*[Title/Abstract])) OR (Endometrioid Adenocarcinoma*[Title/Abstract])) |
| #2 | (((((((((((Contrast-enhanced ultrasound[Title/Abstract]) OR (Contrast enhanced ultrasound[Title/Abstract])) OR (Contrast-enhanced ultrasonography[Title/Abstract])) OR (CEUS[Title/Abstract])) OR (Contrast-enhanced sonography[Title/Abstract])) OR (Enhanced-ultrasonography[Title/Abstract])) OR (Ultrasonic Contrast[Title/Abstract])) OR (Acoustic Contrast[Title/Abstract])) OR (Ultrasonic angiography[Title/Abstract])) OR (Phlebography[Title/Abstract])) OR (Venography[Title/Abstract])). |
| #3 | (#1) AND (#2) |

Table S2 Evaluation of methodological quality of included studies

| **Study** | **Risk of bias** | | | |  | **Applicability** | | |
| --- | --- | --- | --- | --- | --- | --- | --- | --- |
|  | **Patient Selection** | **Index Test** | **Reference Standard** | **Flow and Timing** |  | **Patient Selection** | **Index Test** | **Reference Standard** |
| Ben ^[13]^ | L | U | U | U |  | L | L | L |
| Chen ^[14]^ | L | L | U | L |  | L | L | L |
| Ding ^[22]^ | L | L | L | U |  | L | L | L |
| Du ^[23]^ | L | L | U | U |  | L | L | L |
| Huang ^[24]^ | L | U | U | L |  | L | L | L |
| Huang ^[25]^ | L | U | U | U |  | L | L | L |
| Li ^[26]^ | L | U | U | U |  | L | L | L |
| Lin ^[27]^ | L | L | U | L |  | L | L | L |
| Lu ^[28]^ | L | U | U | U |  | L | L | L |
| Mao ^[29]^ | L | U | U | U |  | L | L | L |
| Ni ^[30]^ | L | U | U | U |  | L | L | L |
| Pei ^[31]^ | L | L | U | L |  | L | L | L |
| Shabiti ^[32]^ | L | U | U | U |  | L | L | L |
| Song ^[33]^ | L | U | U | U |  | L | L | L |
| Su ^[34]^ | L | U | U | U |  | L | L | L |
| Sun ^[21]^ | L | U | U | U |  | L | L | L |
| Tian ^[35]^ | L | U | U | L |  | L | L | L |
| Wang ^[36]^ | L | L | U | U |  | L | L | L |
| Xie ^[37]^ | L | L | U | U |  | L | L | L |
| Xu ^[38]^ | L | U | U | U |  | L | L | L |
| Ye ^[15]^ | L | L | U | U |  | L | L | L |
| Zhang ^[39]^ | L | U | U | U |  | L | L | L |
| Zou ^[40]^ | L | L | U | L |  | L | L | L |

L, Low risk; U, Unclear risk.
